# Spatial variation and inequities in antenatal care coverage in Kenya, Uganda and mainland Tanzania using model-based geostatistics: a socioeconomic and geographical accessibility lens

**DOI:** 10.1186/s12884-022-05238-1

**Published:** 2022-12-06

**Authors:** Peter M. Macharia, Noel K. Joseph, Gorrette Kayondo Nalwadda, Beatrice Mwilike, Aduragbemi Banke-Thomas, Lenka Benova, Olatunji Johnson

**Affiliations:** 1 Population Health Unit, Kenya Medical Research Institute-Wellcome Trust Research Programme, Nairobi, Kenya; 2 Centre for Health Informatics, Computing, and Statistics, Lancaster Medical School, Lancaster University, Lancaster, UK; 3 College of Health Sciences, Makerere University, Kampala, Uganda; 4 Community Health Nursing Department, School of Nursing, Muhimbili University of Health and Allied Sciences, Dar es Salaam, Tanzania; 5 School of Human Sciences, University of Greenwich, London, UK; 6 Department of Public Health, Institute of Tropical Medicine, Antwerp, Belgium; 7 Department of Mathematics, The University of Manchester, Manchester, UK

**Keywords:** Antenatal care, Inequities, Household wealth, Maternal education, Travel time to healthcare, Model-based geostatistics, Subnational, District, Kenya, Uganda, Tanzania

## Abstract

**Background:**

Pregnant women in sub-Saharan Africa (SSA) experience the highest levels of maternal mortality and stillbirths due to predominantly avoidable causes. Antenatal care (ANC) can prevent, detect, alleviate, or manage these causes. While eight ANC contacts are now recommended, coverage of the previous minimum of four visits (ANC4+) remains low and inequitable in SSA.

**Methods:**

We modelled ANC4+ coverage and likelihood of attaining district-level target coverage of 70% across three equity stratifiers (household wealth, maternal education, and travel time to the nearest health facility) based on data from malaria indicator surveys in Kenya (2020), Uganda (2018/19) and Tanzania (2017). Geostatistical models were fitted to predict ANC4+ coverage and compute exceedance probability for target coverage. The number of pregnant women without ANC4+ were computed. Prediction was at 3 km spatial resolution and aggregated at national and *district -level *for sub-national planning.

**Results:**

About six in ten women reported ANC4+ visits, meaning that approximately 3 million women in the three countries had <ANC4+ visits. The majority of the 366 districts in the three countries had ANC4+ coverage of 50–70%. In Kenya, 13% of districts had < 70% coverage, compared to 10% and 27% of the districts in Uganda and mainland Tanzania, respectively. Only one district in Kenya and ten districts in mainland Tanzania were likely met the target coverage. Six percent, 38%, and 50% of the districts had at most 5000 women with <ANC4+ visits in Kenya, Uganda, and mainland Tanzania, respectively, while districts with > 20,000 women having <ANC4+ visits were 38%, 1% and 1%, respectively. In many districts, ANC4+ coverage and likelihood of attaining the target coverage was lower among the poor, uneducated and those geographically marginalized from healthcare.

**Conclusions:**

These findings will be invaluable to policymakers for annual appropriations of resources as part of efforts to reduce maternal deaths and stillbirths.

**Supplementary Information:**

The online version contains supplementary material available at 10.1186/s12884-022-05238-1.

## Background

Despite a 38% reduction in maternal mortality ratio (MMR) between 2000 and 2017, about 810 women died each day due to complications of pregnancy and childbirth in 2017 globally [1]. Similarly, two million stillbirths occurred in 2019, despite a 35% reduction since 2000 [2]. The majority of the maternal deaths (66%) and stillbirths (40%) occurred in sub-Saharan Africa (SSA) [1, 2]. Across the globe, SSA still has one of the highest disease burdens, with an 89-fold higher MMR and a 36-fold higher stillbirth rate compared to Europe. Within SSA, MMR and stillbirths vary between [1, 2] and within countries [3, 4]. This variation has been attributed mainly to inequities in access to quality health services, varying levels of poverty, and differences in education attainment [3–6].

Most maternal deaths and stillbirths are preventable through high-quality care in pregnancy and during and after childbirth [7]. Antenatal care (ANC) is a crucial element of the continuum of care and aims to prepare for birth, prevent, detect, alleviate, and manage pregnancy-related complications that may occur. ANC also presents an opportunity for health promotion among women, families, and communities [8–10].

The World Health Organization (WHO) developed the “*focused ANC model”* in the 1990s to guide routine care at *four* critical times during pregnancy (ANC4+) [11]. This guideline was revised to *eight* contacts in the 2016 update to improve the experience of care and minimize the risk of poor pregnancy outcomes [8, 9]. However, in SSA, the proportion of women who meet even the pre-2016 requirement of four ANC visits remains suboptimal. While eight in ten (81.9%) pregnant women in SSA report at least one ANC visit, only 53.4% had at least four visits in 2020 [12]. In Latin America and the Caribbean, 91% of women had ANC4+ visits [12]. The ANC4+ coverage in Kenya (58.5%), Uganda (56.7%) and Tanzania (62.2%) is moderate relative to other SSA countries like Ghana (90.5%) and Liberia (87.3%) [12, 13]. ANC coverage is also heterogeneous within countries in SSA, with wide coverage gaps by residence (rural and urban), maternal education, and household wealth quintile [14–17].

To reduce maternal and perinatal mortality through ensuring equitable access to ANC services, it is crucial to examine how ANC4+ coverage varies across sub-groups at high spatial resolution [15, 18]. This will inform where and who should be targeted the so-called *hotspots* requiring action. The WHO-led Ending Preventable Maternal Mortality (EPMM) working group outlined global targets and strategies for reducing maternal mortality within the Sustainable Development Goals (SDGs) framework [19, 20]. ANC4+ coverage is one of the core priority indicators within the global monitoring and reporting framework [18]. In this framework, at least 90% of all countries and 80% of all districts in a country are expected to have over 70% (target coverage) of pregnant women having ANC4+ visits by 2025 [19]. We apply this target coverage to guide our exceedance probability analysis. Countries also set local targets; Kenya’s targets ANC4+ coverage of 57% by 2020/21 [21], 50% in Uganda by 2021/22 [22] while Tanzania targeted 60% by 2020 [23]. Tanzania is also tracking early ANC coverage (< 12 weeks) aiming a 60% coverage by 2025 [24]. These countries track the targets monthly using routine data supplemented by survey data when available. However, routine data has poor reporting rates and lacks socioeconomic data for equity analysis [25].

Recognizing that relying on broad, aggregate, and national-level estimates masks inherent spatial pockets of sub-national inequities, countries need to evaluate ANC4+ coverage along sub-groups [18, 26] at high spatial resolution. Previous studies have examined ANC4+ coverage across sub-groups in Kenya, Uganda, and Tanzania [13, 14, 16, 27–30]. However, none of the earlier studies mapped ANC4+ coverage inequities per sub-group at high spatial granularity. Further, previous studies have not assessed the extent to which EPMM’s ANC4+ target coverage has been achieved overall and across subgroups. Model-based geostatistics (MBG) [31] offers a principled likelihood-based approach to problems concerning the modeling of the spatial variation of a phenomenon of scientific interest such as ANC4+ and robustly assesses attainment of target coverage. It has been applied widely across public health problems where the goal is to make inferences using spatially discrete cross-sectional survey data, especially in low resource settings where disease registries are incomplete or non-existent [32–34]. In this study, we aimed to model ANC4+ coverage, likelihood of achieving target coverage and number of women who need to be reached disaggregated by three equity stratifiers (household wealth, woman’s education, and travel time to nearest health facility) using data from household surveys in Kenya, Uganda, and mainland Tanzania. All analyses were at 3 × 3 km spatial resolution and aggregated by district.

## Methods

### Geographic and country context

Kenya, Uganda, and Tanzania are located in East Africa and share national borders (SI Fig. 1). Each country is subdivided into *districts* that are used for healthcare planning, 47 in Kenya (counties), 135 in Uganda (districts) and 184 in mainland Tanzania(councils) (SI Fig. 1). Population, health, socioeconomic and demographic indicators for each country are presented in SI Table 1. The healthcare system in the three countries is decentralized, running a hierarchical referral system from primary to tertiary level health facilities with both public and private health facilities [21, 22, 24]. These health facilities are expected to serve ANC clients through a recommended package of interventions [8, 9, 11]. The health sector financing in the three countries is mainly dependent on funds from the government, donors, and out-of-pocket payments [35–37]. Over time, these countries have put in place policies to make maternal health services, including ANC, affordable and accessible through subsidies, incentives, partial or full removal of user fees, vouchers, conditional cash transfers and insurance programs [29, 38–42]. ANC guidelines monitored ANC4+ coverage at the time of the survey in the three countries [21–24].

**Fig. 1:**
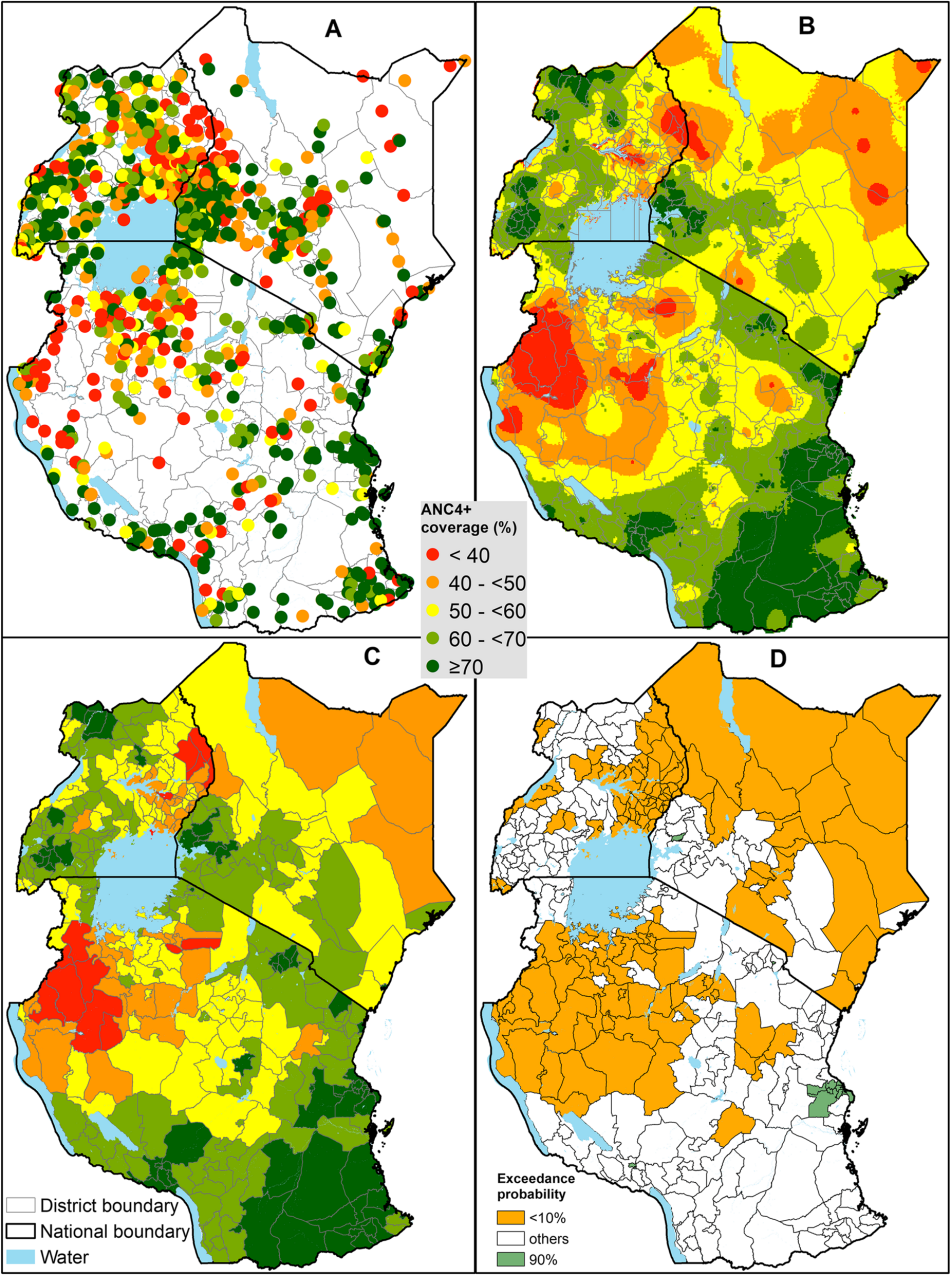
Percentage of pregnant women with at least 4 ANC visits based on the pregnancy preceding their most recent live birth during the 3 years preceding the survey. Empirical observations (**A**), predicted surfaces at 3 km spatial resolution (**B**) aggregated at *district* level (**C**) and exceedance probability for a 70% target in Kenya, Uganda, and Tanzania mainland

### Data

We used data from the most recent nationally representative Malaria Indicator Surveys (MIS) in Kenya 2020 [43], Uganda 2018/19 [44], and Tanzania 2017 [45]. MIS are stand-alone cross-sectional household surveys which collects data on key indicators of malaria and population health, including that of pregnant women. The sampling strategy is detailed in supplementary information 1 (SI) section A2. Our study sample included ANC history of 10,237 women of reproductive age (15–49 years) for their most recent live birth in the 3 years preceding the surveys. The women belong to randomly selected households within sampled enumeration areas (EAs)/clusters. Each cluster is represented by a displaced geographical coordinate to protect respondent confidentiality [46]. Urban and rural clusters are displaced by up to 2 and 5 km, respectively while remaining within boundaries of the district or region considered in the survey. Further, 1% of the rural clusters are displaced by up to 10 km [46].

#### Study variables

The outcome variable was the percentage of women who reported receiving ANC4+ visits. Women were asked how many visits they received during pregnancy, and during those visits, to list all types of health providers/professionals they saw. We defined doctors, nurses, midwives, medical assistants, clinical officers, assistant clinical officers, assistant nurses, maternal and child health aides as qualified health professionals for the purpose of ANC provision. Women reporting ANC visits but not listing at least one of these providers were categorized as not receiving ANC. Although the study surveys were conducted during the first phase of implementing the new WHO recommendation of at least eight ANC contacts (ANC8+), none of the three countries had transitioned to the ANC8+ model at the time of data collection or had explicit policy targets for its coverage [21–24]. Further, the observed ANC8+ coverage based on the study surveys was very low (3.5% in Kenya, 1.4% in Uganda, and 1.2% in mainland Tanzania) insufficient for robust geostatistical modelling at high spatial resolution. As such, analyses in this study were based on the previous WHO recommendation of ANC4+ and in line with the EPMM targets [11, 19]. Study variables were based on factors known to influence ANC use [47–49] and data available from the three MIS (Table 1).

**Table 1:** The outcome and covariates based on Malaria Indicator Survey in Kenya (2020), Tanzania (2017) and Uganda (2018/19). Travel time was modelled while nighttime lights were derived from satellite imagery. Geographical coordinates were available at the cluster level, and all data were resolved at this level

Category	Variable	Description
Outcome	ANC4+	At least four antenatal care visits by a skilled provider (doctors, nurses, midwives, medical assistants, clinical officers, assistant clinical officers, assistant nurses, maternal and child health aides collapsed to either 0 (< 4 ANC visits, including no visits) or 1 (ANC4+ visits).
Aggregation unit	District	Health planning subnational units for each country, 47 counties in Kenya, 135 districts in Uganda, and 184 districts in mainland Tanzania (S1 Fig. 1) which were used as the aggregation unit across the equity stratifiers.
Equity stratifiers	Maternal education	The highest level of education attained by the woman at the time of the survey re-classified as no education (0) or some education (primary, secondary, or higher, whether complete or incomplete) (1)
	Household wealth	Relative household wealth classified into quintiles based on principal component analysis of household assets and other characteristics [50]. The poorest and poor quintiles were collapsed to *poor* (0), while middle, rich and richest collapsed to *non-poor* category (1).
	Travel time to nearest health facility	Travel time in minutes to the nearest health facility analytically modelled (continuous variable) and extracted for each survey cluster. It was categorized as having good access (≤1 hour) or being marginalized (> 1 hour) [51].
Other covariates	Nighttime light lights (NTL)	Remotely sensed nighttime light emissions based on satellite observations expressed using radiance (watt per steradian per square meter). Mean NTL was extracted per cluster as a continuous variable.
	ANC initiation for women with at ≥1 visit	Initiation of first ANC visit for women with at least 1 visit during pregnancy classified as timely (1) (visit within first trimester) or late (0) (visit during the second or third trimester). Those 0 visits were treated as missing
	Health media exposure	Any form of exposure to health information (from radio, internet, magazines, social media, newspapers, social mobilization campaigns by the government, billboards, television, or community events) (1) or no access at all (0). We considered any form of access to health information (exposure) instead of frequency of access to general information from media that is not health nuanced. The sources of health-related messages in Kenya were 14 while Uganda and Tanzania had eight sources.
	Decision to seek ANC services	Woman’s participation in decision-making to seek ANC services for herself classified as *involved* (made the decision independently, or joint decision with spouse) or *never involved* (decision made by either the spouse or someone else). This is based on the household structure and who was living with the woman at the time of the survey.
	Birth order	The ordinal position of the index live birth during the survey classified as first birth or higher order birth.

Two factors not sourced from the MIS were nighttime lights (NTL) and travel time to the nearest health facility. NTL is a proxy for urbanization, gross domestic product, population density and economic activity [52, 53]. Its inclusion alongside other covariates (Table 1) correlated with the urban/rural clusters in geostatistical models for disease mapping accounts for the sampling design implicitly [54, 55]. Annual NTL, temporally matched to survey year, produced using monthly cloud-free radiance averages, made from low light imaging day/night band data collected by the NASA/NOAA Visible Infrared Imaging Radiometer Suite was used [56]. We extracted NTL per cluster within a buffer to minimize the effect of displaced cluster coordinates in ArcMap version 10.5 (ESRI Inc., Redlands, CA, USA).

We modelled travel time to the nearest health facility (spatial access) using approaches that combine several modes of transport in a single journey [57, 58] based on a least-cost path algorithm implemented in AccessMod software alpha version 5.7.8 (WHO, Geneva, Switzerland) [59]. We accounted for the road network, land use, topography, and transport barriers except where a road intersected a barrier [57–59]. We leveraged the SSA master health facility list (MHFL) comprising public health facilities managed by the government, local authority, faith-based and non-governmental organizations capable of offering ANC [57, 60, 61]. The SSA MHFL reflects facilities available around 2015–2018. However, the Kenyan list had been updated (2020) by incorporating data from Kenya’s routine data reporting system and Kenya’s MHFL [62]. We extracted the mean travel time for each cluster as done for the NTL gridded surfaces.

#### Equity stratifiers

Equity stratifiers were based on factors known to influence ANC4+ coverage, within EPMM recommendations, based on data availability and in WHO’s list of the main barriers to receiving or seeking care during pregnancy [7, 18, 19, 26, 47–49]. They included maternal education, household wealth and travel time to the nearest healthcare facility and were stratified as shown in Table 1. The stratification followed a pragmatic approach, with a policy interpretation, supported by literature and ensuring each arm had a considerable number of observations to allow for robust inference using MBG. Districts were then used as the unit of aggregation.

#### Missing data

Data on maternal autonomy (decision to seek ANC services) were only collected on the Kenya MIS, while data on ANC initiation was not reported on the Tanzania MIS. Women who attended ANC but had a *“don’t know”* response for the number of ANC visits or when they initiated their first visit were recoded as missing (1.4% in Kenya, 0.6% in Uganda, and 2.1% in mainland Tanzania). However, the three variables with missing data did not exceed 2.1% of the total sample size by country and were excluded from the analysis (SI section A2).

### Geostatistical modeling

#### Spatial exploratory analysis and model selection

Exploratory analysis is the first stage of geostatistical analysis [54]. It entails visualizing the spatial distribution of sampled clusters (Fig. 1A), examining the correlation between covariates, assessing the relationship between ANC4+ and covariates, and testing for residual spatial correlation [54]. We undertook these steps as detailed in SI section A3. Briefly, Pearson’s correlation was implemented in corrplot package in R [63] while empirical logit [64] was used to assess the association between ANC4+ coverage and the covariates and visualized with scatter plots. To select a set of parsimonious predictors used as fixed effects during geostatistical modeling, we used a non-spatial generalized linear model relating the covariates with ANC4+ coverage. The selection was done by country and equity stratifier resulting in 21 models. Finally, we assessed the evidence of spatial correlation after accounting for fixed effects (parsimonious predictors) through an empirical variogram (S1 Section A3).

#### Parameter estimation and spatial prediction

Separate Bayesian geostatistical models were used to model ANC4+ coverage for each country and equity strata. Each model contained explained factors (fixed effect) and unexplained factors (random effect). The fixed effect was modelled using the predictors denoted as *d*^'^(*x*)*β*, where *d*(*x*) is the vector of parsimonious predictors with the corresponding coefficient *β*. The random effect was modelled using two terms, *S*(*x*) to account for the spatial residual variation and Z to account for the measurement error or small-scale variation that is not captured in *S*(*x*). Specifically, the variation in ANC4+ coverage *P*(*x*) at location x was modelled using a binomial geostatistical model (Eq. 1).

1 $$\log\left\{\frac{P(x)}{1-P(x)}\right\}=d'(x)\beta+S(x)+Z$$

*S*(*x*)was modelled as a zero-mean discretely indexed Gaussian Markov Random Field (GMRF) with Matérn correlation function [65]. All fixed and random effect parameters were estimated using the integrated nested Laplace approximation (INLA) and Stochastic Partial Differential Equation (SPDE) implemented in INLA package [65, 66]. Prediction of ANC4+ coverage was obtained using the simulation from posterior distributions of all the parameters and summarized using the mean, standard error and 95% confidence interval (CI) at 3 × 3 km spatial resolution. The high-resolution surfaces were aggregated by district. Additional details about geostatistical models are provided in SI section A4.

We assessed the likelihood (exceedance probability-EP) that each pixel and district had ANC4+ coverage above 70%, the target coverage based on EPMM strategy [19] (SI section A5). An EP value close to 100% indicates that ANC4+ coverage is highly likely to be above the target; if close to 0%, ANC4+ coverage, is highly likely to be below the target; if close to 50%, ANC4+ coverage, is equally likely to be above or below the target.

#### Model validation

We validated our models by checking if the fitted correlation function was compatible with the data using a variogram-based procedure [67, 68] detailed in SI section A6. It entailed simulating many variograms from the fitted model and then comparing them with the estimated empirical variogram from the data. We concluded that the adopted correlation function is compatible with our data if the estimated empirical variogram lies entirely in the 95% confidence interval of the simulated empirical variograms.

#### Computing the number of women with ANC4+ and < ANC4

We estimated the number of pregnant women with ANC4+ visits by multiplying the 3 km gridded surfaces showing ANC4+ coverage from geostatistical models and population gridded surfaces of pregnant women obtained from the WorldPop portal [69]. The number of pregnant women with fewer than four visits (<ANC4+) was obtained by subtracting those with ANC4+ visits from the total number of pregnant women. The results were aggregated by country and district. Briefly, to construct the population density maps, mid-year population of under 1 year (corrected for mortality and migration) were extrapolated by Worldpop based on United Nations (UN) data on births and WorldPop’s estimates of children under 1 year to estimate total annual births. The births were adjusted to match the UN total births by country. The Guttmacher birth to pregnancy rate was used to compute the number of annual pregnancies. Gridded pregnancy surfaces were available for 2020 in Kenya and 2017 for Uganda and mainland Tanzania at 1 km spatial resolution [69].

STATA (StataCorp. 2015. *Stata Statistical Software: Release 14*. College Station, TX: StataCorp LP.) was used for descriptive analysis, R statistical software [70] for geostatistical modelling and ArcMap version 10.5 (ESRI Inc., Redlands, CA, USA) for all cartographies.

## Results

### Characteristics of study participants and model development

Our study sample included ANC history of 2036 women in Kenya, 3840 in Uganda and 4361 in mainland Tanzania for their most recent live birth in the 3 years preceding the surveys. The descriptive summary of the socioeconomic and demographic characteristics of these women are presented in Table 2. The percentage of women with some form of education was high and ranged from 78.3% (mainland Tanzania) to 89.9% (Kenya). Those from poor and poorer wealth quantiles ranged from 38.7% in Kenya to 50.2% in Uganda. Uganda had the highest percentage of women living outside a one-hour catchment area of the nearest public health facility (20.9%), followed by mainland Tanzania (15.4%), and Kenya (7.3%). Exposure to health-related knowledge was high in Kenya (90.0%), relative to Uganda (37.4%) and mainland Tanzania (47.5%) and women in Kenya reported a wider a variety of sources of such information. Model building results are presented in S1 Sections A3, A4, A5, A6, and A8. The validity of the adopted spatial structure of each geostatistical model showed that the assumed spatial correlation function was compatible with our data.

**Table 2:** Socioeconomic and demographic characteristics of women based on the pregnancy preceding their most recent live birth in the 3 years preceding Malaria Indicator Survey in Kenya 2020, Uganda 2018/19, and mainland Tanzania 2017

Variable	Categories	Kenya ***N*** = 2036	Uganda ***N*** = 3840	mainland Tanzania ***N*** = 4361
		*N (weighted percentage: 95% CI)*		
ANC4+	Yes	60.8: 57.0–64.5	56.4: 53.8–58.9	60.9: 57.9–63.7
	No	39.2: 35.6–43.0	43.6: 41.1–46.2	39.1: 36.3–42.1
Maternal education	Some education	89.9: 85.3–93.2	82.6: 79.3–85.5	78.3: 75.9–80.6
	No education	10.1: 6.8–14.7	17.4: 14.5–20.7	21.7: 19.4–24.1
Household wealth	Poor (two poorer quintiles)	38.7: 32.0–45.9	50.2: 44.9–55.6	46.1: 41.6–50.8
	Non-poor (three wealthier quintiles)	61.3: 54.1–68.0	49.8: 44.4–55.1	53.9: 49.2–58.4
Decision to seek ANC services	Woman involved	89.3: 86.6–91.6	–	
	Woman not involved	10.7: 7.6–14.7)		
Birth order	First birth	31.0: 27.3–35.0	19.4: 17.0–21.9	23.3: 21.1–25.6
	High order births	69.0: 65.0–72.7	80.6: 78.1–82.9	76.7: 74.4–78.9
Health media exposure	Yes	90.0: 87.2–92.2	37.4: 34.6–40.3	47.5: 45.1–49.9
	No	10.0: 7.8–12.8	62.6: 59.7–65.4	52.5: 50.0–54.9
Travel time to nearest public health facility	Within 1 hour	92.7: 87.9–95.7	79.1: 72.5–84.5	84.6: 79.3–88.8
	Outside 1 hour	7.3: 4.3–12.1	20.9: 15.5–27.5	15.4: 11.2–20.7
ANC initiation among women with ≥1 visit	First trimester	30.0: 26.1–34.1	34.3: 31.5–37.1	–
	Second/third trimester	70.0: 65.9–73.9	65.7: 62.9–68.5	

### National coverage of ANC4+ visits

Approximately six in ten pregnant women had at least 4 ANC visits, 60.8% (95% CI: 57.0–64.5) in Kenya, 56.4% (53.8–58.9) in Uganda and 60.9% (57.9–63.7) in mainland Tanzania (Table 2). At the national level, none of the countries had achieved the 2025 EPMM ANC4+ target coverage of 70%. However, all the countries had attained their local targets in the survey year, 57% in Kenya by 2020/21, 50% in Uganda by 2020/21 and 60% in Tanzania by 2020. The computed ANC4+ coverage translated to circa 1,362,295 [1,074,933 – 1,626,559] pregnant women in Kenya (2020), 1,378,033 [1,021,299 – 1,708,417] in Uganda (2017) and 1,831,845 [1,360,602 - 2,257,962] in mainland Tanzania (2020). While the percentage of women with ANC4+ visits at the national level was similar, however, due to the different numbers of pregnant women in each country, the number of women with <ANC4+ visits was variable. It ranged from 833,936 [569,672-1,121,298] in Kenya, 982, 535 [652,151-1,339,269] in Uganda to 1,134,884 [708,768-1,606,128] in mainland Tanzania.

### Pixel- (3 km) and district- level coverage of ANC4+ visits

Within each country, we found high evidence of spatial heterogeneity in ANC4+ coverage, ranging from 10% to over 95% of women by survey cluster (Fig. 1A) and by 3 km pixels (Fig. 1B). Large parts of northern Kenya, north-western Tanzania (around Lake Victoria) and eastern Uganda had low coverage of ANC4+ (< 50%) compared to the rest of areas in the three countries at pixel level. Conversely, western Kenya (shores of Lake Victoria), southern Tanzania (bordering Mozambique and along the Indian ocean), and parts of northern and southern Uganda had high ANC4+ coverage (over 70%) relative to other parts of the three countries (Fig. 1B).

When the gridded surfaces (Fig. 1B) were aggregated by district (Fig. 1C), overall, 19% (70 out of 366) of all districts had ANC4+ coverage of over 70%. Thirteen percent (6) of counties in Kenya had > 70%, compared to 10% (13) of districts in Uganda and 27% (70) of the districts in mainland Tanzania. Kenya (57% by 2020/21), Uganda (50% by 2020/21) and Tanzania (60% by 2020), had 75, 78 and 61% of districts with ANC4 coverage greater or equal to their local target (Fig. 1C). Additionally, 62 districts across the three countries had ANC4+ coverage of less than 50%: 30 in Uganda, five in Kenya (Garissa, Marsabit, Wajir, Mandera and West Pokot counties), and 27 in mainland Tanzania (Fig. 1C). Only eight districts (Urambo, Itilima, Kasulu, Biharamulo, Kaliua, Kibondo, Kakonko and Bukombe) in mainland Tanzania had ANC4+ coverage of less than 40%. Among the 27 districts in Uganda with coverage lower than 50%, six (Nabilatuk, Moroto, Pallisa, Buvuma Napak and Amudat) districts had the lowest coverage of less than 40% (Fig. 1C).

The results of spatially overlaying population distribution maps with ANC4+ coverage is shown in SI Fig. 18 by district in the three countries. Three (6.4%), 51 (37.8%), and 93 (50%) districts each had at most 5000 women with <ANC4+ visits in Kenya, Uganda, and mainland Tanzania, respectively. On the hand, 18 (38.3%) districts in Kenya had over 20,000 pregnant women with <ANC4+ visits and only two districts in Uganda (Wakiso and Kampala) and three districts (Kasulu, Kaliua and Geita) in mainland Tanzania (SI Fig. 3). There are five outlier districts with over 30,000 pregnant women having <ANC4+ visits in Kenya (Garissa, Wajir, Mandera, Nairobi and Nakuru counties), two in Uganda (Wakiso and Kampala), and none in mainland Tanzania (SI Fig. 3). Garissa, Wajir and Mandera counties in Kenya had the lowest ANC4+ coverage and a high number of women not receiving ANC4+. In addition, Nairobi and Nakuru counties (Kenya) and Wakiso and Kampala districts (Uganda) had a high number of women not receiving ANC4+ despite moderate ANC coverage, due to their high population.

The likelihood of attaining EPMM ANC4+ target coverage of > 70% on the district level with a high likelihood was suboptimal. No districts in Uganda are likely to have met the threshold with a 90% likelihood (Fig. 1D). However, one county (Vihiga) in Kenya and ten districts in mainland Tanzania met this threshold (Fig. 1D). Among the ten districts in mainland Tanzania, five were in Dar es Salaam region (Ubungo MC, Temeke, Ilala, Kinondoni, and Kigamboni) and three districts (Kibaha urban, Kibaha, and Kisarawe) were in the adjacent Pwani region. Conversely, the poorly performing districts, with the least likelihood (< 10%) of attaining the recommended target, were the majority. They covered northern and south-east Kenya, eastern Uganda, and north-western Tanzania (Fig. 1D).

### ANC4+ coverage by equity stratifiers

**Fig. 2:**
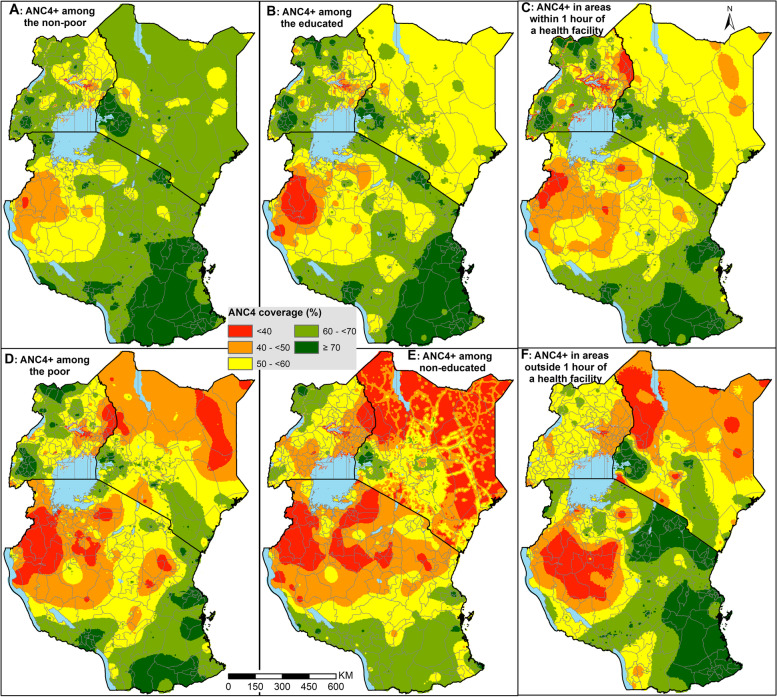
Proportion of pregnant women with at least 4 ANC visits based on the pregnancy preceding their most recent live birth in the 3 years preceding the survey disaggregated by wealth quintile (**A**), maternal education (**B**) and spatial accessibility to care (**C**) in East Africa at 3 × 3 km spatial resolution

**Fig. 3:**
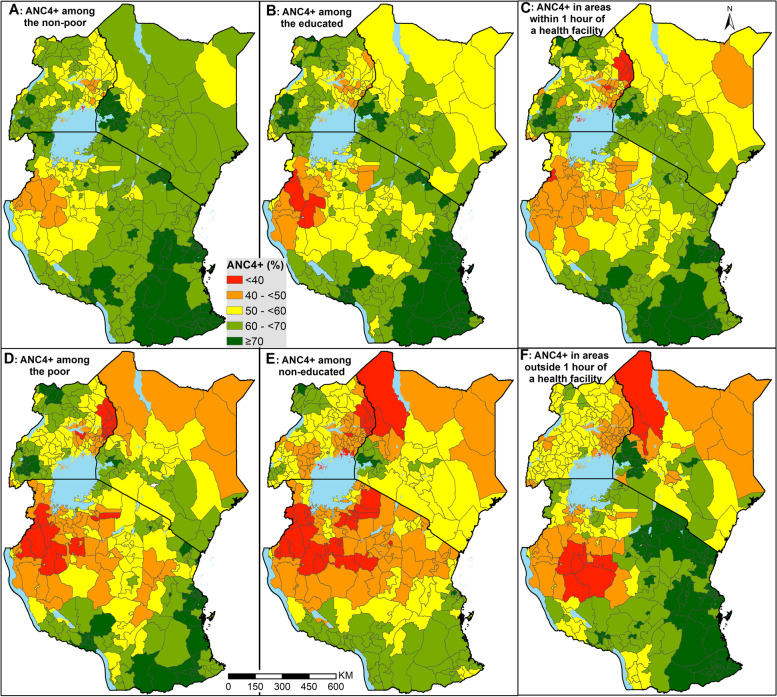
Percentage of pregnant women with at least 4 ANC visits based on the pregnancy preceding their most recent live birth in the 3 years preceding the survey disaggregated by household wealth quintile (**A**), education (**B**) travel time to the nearest facility and (**C**) districts

The estimates presented so far characterize overall coverage among pregnant women without considering sub-groups which might mask disparities. ANC4+ coverage among all the equity stratifiers is shown at pixel-level resolution in Fig. 2 and district level in Fig. 3. The corresponding exceedance probabilities at district level are shown in SI Fig. 14. ANC4+ coverage in each stratifier was highly heterogeneous (Fig. 2), with the general spatial variation following that of the overall coverage (Fig. 1). Overall, ANC4+ coverage among the poor (Fig. 2D), un-educated (Fig. 2E) and marginalized from healthcare access (Fig. 2F), was lower compared to the non-poor (Fig. 2A), educated (Fig. 2B) and those within 1-hour of the nearest health facility (Fig. 2C).

Broadly, ANC4+ coverage per district favored the non-poor, the educated, and those living within 1 h of a public health facility (Figs. 2 and 3). Eighty-one (22%) districts had an ANC4+ coverage of over 70% among the educated (Fig. 3B) and only 6 (2%) districts among the non-educated (Fig. 3E), a 13.5-fold difference in the three countries. Similar findings were observed for household wealth (Figs. 2A, D, 3A and D) and travel time to nearest health facility (Figs. 2C, F, 3C and F). Kenya had only one county with an ANC4+ coverage of less than 50% among those with good access (Fig. 3C) compared to 14 counties among the geographically marginalized from healthcare (Fig. 3F) with a similar trend in Uganda and mainland Tanzania.

Irrespective of the household wealth quintile, maternal education status or proximity to healthcare, districts that met EPMM target coverage with a high likelihood were fewer (SI Fig. 14). Further, among the few districts which attained the target coverage with greater than 90% certainty, the majority were among the non-poor, educated and those living closer to their nearest health facility, while the districts unlikely to have met the target (less than 10% certainty) were mainly among the poor, uneducated and those geographically marginalized from healthcare*.* For example, districts with the poor and uneducated women, there were no districts likely to have met the target coverage across three countries with a high likelihood (SI Fig. 14).

Finally, across all the stratifiers, there is a remarkable pattern of districts with a three-fold burden. That is, intersectionality of vulnerable districts where the same districts has low coverage of ANC4+ among the poor, uneducated and those marginalized from the nearest health facility. These include districts in northern Kenya, eastern Uganda, and north-western Tanzania. Similarly, districts in western Kenya, southern Tanzania, and some parts of northern and southern Uganda had systematic high coverage in all stratification arms.

## Discussion

Monitoring ANC4+ coverage and associated inequities requires quantifying and describing the coverage across population groups defined along socioeconomic and geographic equity lines within countries [19, 20]. This should be at a high resolution, the so-called precise public health [71], to highlight hotspots areas within a country. Our findings show that ANC4+ coverage was moderate, with six in every ten pregnant women reporting having received at least four ANC visits in the three East African countries. At the national level, this is short of the 70% coverage anticipated to be achieved by 2025 under the EPMM strategy. However, national targets set by the governments of each of the three countries were achieved. Compared to similar national estimates about decade ago (since 2021), there have been slight improvements. In the early 2010s, between four and five in ten pregnant women had ANC4+ visits - that is, 47.1% in Kenya (2009), 47.6% in Uganda (2011) and 42.8% in Tanzania (2010) [13]. These improvements may be explained by the concerted efforts of stakeholders which included healthcare investment focused on access, training health professionals, decentralized health care, maternal health education, user fees reduction or abolishment among other targeted initiatives [72–80].

However, despite the moderate national improvements and associated efforts, the current ANC4+ coverage is inequitable, and falls short of recommended levels. Yet, the role of ANC in preventing, detecting, alleviating, and managing pregnancy-related complications that might lead to maternal deaths and perinatal mortality is well known. Our findings show the specific districts that have the least coverage and the linked inequities dragging the coverage. This will aid in targeted allocation of resources, subsequent monitoring and evaluation, and benchmarking. This aligns with the SDG mantra of leaving no one behind and starting with the farthest behind, first. The high-resolution maps in Fig. 2 aid in identifying *hotspots* within the districts with poor coverage, while the exceedance probabilities minimize the chance of misclassifying districts and pixels. This ensures persistent foci of low coverage are correctly identified such that resources are not wasted on interventions and populations who do not require them. We have provided all the district estimates in Additional file 2 for use by policymakers.

The most left behind (lower levels of ANC4+ coverage) districts bore a treble burden where the poorest, with the least education and geographically marginalized from healthcare reside. Women from these districts maybe at a higher risk of maternal mortality and perinatal deaths. There were also districts that had both lowest coverage of ANC4+ and at the same highest number of pregnant without ANC4+ visits. Certainly, resources, and infrastructure are concentrated in wealthier urban places and are scant in poorer and remote areas [81]. The *hotspot* districts and most in need, include West Pokot, Wajir, Mandera, Turkana, Baringo, Garissa, Elgeyo-Marakwet, Marsabit and Trans Nzoia mainly northern Kenya; Amudat, Moroto, Napak, Nabilatuk, Nakapiripirit, Kalangala, Buvuma, Namayingo, Napaka and Palissa majorly located in eastern Uganda and finally, Kakonko, Biharamulo, Kaliua, Kibondo, Bukombe, Chato, Bariadi TC, Urambo, Nzega, Igunga and Itilima mainly north-west Tanzania.

The hotspot counties in northern Kenya have been historically marginalized, are predominately arid and semi-arid and sparsely populated. The region has poor infrastructure, often stricken by conflict and insecurity which may lead to poor geographic access to healthcare. Further, women in this region have low education attainment, mainly come from poor households, and practice some cultural beliefs antagonist to western medical practices [14, 82, 83]. Likewise, eastern Uganda is among the poorest region in the country and has poor coverage of other maternal and child health indicators [28, 84, 85]. Long distances, poor roads and high transport costs, poor services at the health facilities and lack of access to health-related information also impede women to utilize maternal services in this region [86]. Similar situation exists in North-western Tanzania which is poor and has low conditional probability of transitioning from poor to non-poor status [87]. Further, socio-cultural beliefs, distance, lack of transport, perceived poor quality of ANC services have been reported as barriers to ANC use in this region [88]. Combined in the three countries, these factors provide insights on how to improve the poor coverage in the hotspots. However, our study was concerned with identification of these hotspot through predictive modelling [54], therefore, granular (detailed and context-specific) quantitative and qualitative studies should be conducted to better understand why the districts have been left behind.

Our results showed that the poor had lower ANC4+ coverage. It’s the poor who have the highest disease burden, reduced access to healthcare services and the majority do not utilize health services at all [89]. The pro-rich inequities have been observed before [30] and continue to be persist even among the poor pregnant women who are beneficiaries of government initiatives to improve ANC uptake [80, 81, 89]. Ensuring sufficient and timely reimbursements to prevent out-of-pocket payments and minimizing indirect costs of transport [75, 76, 90] will likely increase uptake among the poor ANC clients where initiatives already exist. It is the poor ANC beneficiaries of initiatives who are negatively affected by stock-outs, dysfunctional medical equipment, shortage of healthcare workers, strikes and discrimination [29, 89] since they cannot afford paying services in the private sector. These bottlenecks require addressing so that the woman who have been *left behind* can benefit from programs and initiatives put into place. The high ownership of mobile phones in East Africa can be leveraged to create mobile health program simultaneously with community health workers (CHWs) to facilitate follow-ups and minimize socioeconomic barriers [91] among the poor. Determining the degree of follow-up needed based on ANC user characteristics during the first ANC visit can also be used to increase return visits and ANC uptake.

Women without formal education had lower ANC4+ coverage. Maternal education and household wealth and are linked. Women from poor households often have lower educational attainment which negatively affects utilization [92] as observed in the hotspot districts. In the short run, health promotion and outreach campaigns among pregnant will be useful [91, 93] at the village-level [93] or through mass media [94] in the hotspots. This could neutralize harmful traditions and cultural beliefs, misinformation from family or traditional healers, or cases where pregnant women are misled to delay ANC visits [84, 95]. There is a need to raise awareness about new initiatives meant to increase uptake of ANC since lack of awareness has been a barrier in previous initiatives [38, 77, 96]. There is a necessity to integrate and bolster the need for maternity care seeking into educational curriculum. In the long term, higher education attainment will be vital in increasing women’s autonomy, improved access to healthcare information, and may lead to higher socioeconomic status [97] in the hotspot areas.

Long travel time remains a challenge among women in remote areas even where interventions have been implemented [90, 98] and has been linked with lack of public transport and roads in poor conditions [89, 99–101]. Access to bicycles has shown to be a pro-poor option in increasing access to health centers and can be used as entry point to intervene on areas with poor geographical access [100], supplemented with contracted transporters [77]. Mobile services could also be implemented to meet the women in their communities [14]. Under the Beyond Zero campaign in Kenya, mobile clinics have provided healthcare to poor and marginalized communities [102]. CHWs are integral in promoting maternal care seeking [103] and might be effective in the hard-to-reach areas [104].

Beyond the demand side challenges, there is also a need to strengthen the supply side to guard against inadequate drugs, equipment, infrastructure, skilled human resources, overburdened health facilities, longer waiting times, reduced health worker motivation and quality of care [38, 72, 75–77, 90, 96]. Further, coverage might have been affected by the COVID-19 pandemic, health workers strikes and absenteeism which were associated with a lower likelihood of attending ANC [105–107]. The poor usually bear the burden since they rely mainly on the public sector and cannot afford care from the private sector [108, 109]. The pandemic strained the health system, disrupted essential health services due to inability to access healthcare, transport restrictions, curfew, and fear of contracting the virus when seeking care [110].

## Strengths and limitations

The key strengths of our study lie in deriving high resolution maps per each equity stratum, unlike previous studies and if they do, the resolution is course and unsuitable for granular targeting and prioritization. Notable effort is STATcompiler by the DHS program [13] that produces similar estimates as our study and make it publicly available, however, they disaggregate at broad administrative regions. We have also used exceedance probabilities to account for the uncertainty in the data and quantified the likelihood of meeting target ANC4+ coverage, an aspect that has not been considered in previous ANC4+ coverage studies. Another strength is the use of nationally representative surveys which makes our findings to be comparable and generalizable.

Despite the strengths of our study, there are some limitations. There might have been recall bias synonymous with any retrospective data. There was also selection bias since the surveys included women with a live birth 3 years preceding a survey. Women who might have died during pregnancy or with other birth outcomes were excluded. Related to this is the population data that represented all pregnancies; however, ANC visits were asked only when those pregnancies resulted in live births. The conceptual discrepancy might have biased the estimated number of women with ANC4+ visits. The surveys were conducted at different time points across the three countries - Kenya (2020), Uganda (2018/19) and Tanzania (2017)- limiting temporal comparisons between the countries.

The displacement of cluster coordinates due to confidentiality was not accounted for but was minimized by taking averages of estimates within a buffer. Factors that are associated with ANC beyond those collected during the MIS were not considered except for travel time and NTL. We assumed pregnant women used their nearest facility, yet some proportion bypass their nearest facility [111]. We also did not account for weather variation, traffic jams and other factors that affect transport when estimating travel time. Further, having geographical access is not equivalent to either use of care nor its high quality [112]. We used the number of ANC visits with a qualified professional but did not incorporate data on the content or quality of this care, which is critical to the effectiveness of ANC as a maternal and perinatal mortality reduction strategy. We focused on ANC4+ coverage, however, timing of first visit is also critical to achieving four visits. Women who start late, have very low likelihood of reporting ANC4+ visits, which merits examination in a similar way as we did for ANC4 + .

Household surveys provide an opportunity to monitor the coverage, however, they are conducted every 3 to 5 years, limiting tracking at a higher temporal granularity. In addition, sample size from surveys is often limited and inadequate for high spatial resolution risking a covariate driven ANC4+ coverage [113] especially when stratified as we did. On the other hand, routine health data offer an alternative source of information to monitor ANC4+ coverage. However, routine data are limited due to poor reporting rates, challenges in determining accurate catchment population [25] and does not collect socioeconomic datasets relevant to equity assessment. However, routine data can be linked on spatially smoothed equity stratifiers from household surveys and used for equity monitoring. Finally, despite the findings, we cannot infer causality with the cross-sectional survey data that we used.

## Conclusions

ANC coverage rates have remained moderate, with about 60% of pregnant women having the recommended four or more visits provided by skilled health personnel in East Africa. The likelihood of attaining district-level target coverage by 2025 is very low. Further, the coverage is inequitable, with women from poor households, without formal education and geographically marginalized from formal healthcare having persistently lower coverage and lower likelihood of receiving at least four visits. The spatially disaggregated information will be valuable to policymakers for improved targeting of annual appropriations and leveraging initiatives aiming to improve coverage of recommended interventions and reducing maternal and perinatal mortality.

## Supplementary Information

**Additional file 1: Section A1.** Key indicators for Kenya, Uganda, and mainland Tanzania. **SI Fig. 1.** Health planning units in Uganda, Kenya, and Mainland Tanzania. **SI Table 1.** Key indicators for Kenya, Uganda, and Tanzania. **Section A2.** Sampling in malaria indicator surveys. **SI Table 2.** Proportions of missing observations. **S1 Section A3.** Exploration of the relationship between the prevalence and covariates. **S1 Fig. 2.** Correlation plot for Kenya. **SI Fig. 3.** correlation plot for Tanzania. **SI Fig. 4.** Correlation plot for Uganda. **S1 Fig. 5.** Relationship between the empirical coverage of ANC4+ and the predictors for Kenya. **S1 Fig. 6.** Relationship between the empirical coverage of ANC4+ and the predictors for Tanzania. **SI Fig. 7.** Relationship between the empirical coverage of ANC4+ and the predictors for Uganda. **SI Fig. 8.** Empirical variogram for Kenya. **SI Fig. 9.** Empirical variogram for Uganda. **SI Fig. 10.** Empirical variogram for Tanzania. **SI Section A4.** Parameter estimation and spatial prediction. **SI Fig. 11.** Kenya’s triangulated mesh to build the SPDE model. **SI Fig. 12.** Uganda’s triangulated mesh to build the SPDE model. **SI Fig. 13.** Tanzania’s triangulated mesh to build SPDE model. **SI Section A5.** Exceedance probabilities. **SI Fig. 14.** Exceedance probability for Kenya, Uganda, and mainland Tanzania. **SI Section A6.** Validating the assumed spatial correlation function. **SI Fig. 15**. Empirical variogram estimated from the mixed effect model, including the 95% confidence interval band obtained from a simulation from the fitted model in Kenya. **SI Fig. 16.** Empirical variogram estimated from the mixed effect model, including the 95% confidence interval band obtained from a simulation from the fitted model in Uganda. **SI Fig. 17.** Empirical variogram estimated from the mixed effect model, including the 95% tolerance band obtained from a simulation from the fitted model in Tanzania. **Fig. 18.** The absolute number of women with less than 4 ANC visits across health planning units in Uganda, Kenya, and mainland Tanzania. **SI section A8.** Parameter estimates and corresponding 95% credible interval.

**Additional file 2:** District level estimates for ANC4+ across equity stratifiers.

## Data Availability

The full database of sample household surveys that supports the findings of this study for Kenya, Uganda and Tanzania is available open access from DHS program data portal available to registered users at https://dhsprogram.com/data/available-datasets.cfm.

## Abbreviations

AIC: Akaike information criterion
ANC: Antenatal care
EA: Enumeration areas
EP: Exceedance probability
EPMM: Ending Preventable Maternal Mortality
HIC: High income countries
INLA: Integrated nested Laplace approximation
KMIS: Kenya MIS
LIC: Low-income countries
MBG: Model-based geostatistics
MIS: Malaria Indicator Survey
NTL: Nighttime Lights
SDGs: Sustainable Development Goals
SSA: Sub-Saharan Africa
SPDE: Stochastic Partial Differential Equation
TMIS: Tanzania MIS
UMIS: Uganda MIS
WHO: The World Health Organization
