# Supplementary material for: Spatial variation and inequities in antenatal care coverage in Kenya, Uganda and mainland Tanzania using model-based geostatistics: a socioeconomic and geographical accessibility lens
Source: BMC Pregnancy Childbirth. 2022 Dec 6;22:908. doi: 10.1186/s12884-022-05238-1 (PMC9724345; doi:10.1186/s12884-022-05238-1)
Supplement: Supplementary file 1 — Additional file 1: Section A1. Key indicators for Kenya, Uganda, and mainland Tanzania. SI Fig. 1. Health planning units in Uganda, Kenya, and Mainland Tanzania. SI Table 1. Key indicators for Kenya, Uganda, and Tanzania. Section A2. Sampling in malaria indicator surveys. SI Table 2. Proportions of missing observations. S1 Section A3. Exploration of the relationship between the prevalence and covariates. S1 Fig. 2. Correlation plot for Kenya. SI Fig. 3. correlation plot for Tanzania. SI Fig. 4. Correlation plot for Uganda. S1 Fig. 5. Relationship between the empirical coverage of ANC4+ and the predictors for Kenya. S1 Fig. 6. Relationship between the empirical coverage of ANC4+ and the predictors for Tanzania. SI Fig. 7. Relationship between the empirical coverage of ANC4+ and the predictors for Uganda. SI Fig. 8. Empirical variogram for Kenya. SI Fig. 9. Empirical variogram for Uganda. SI Fig. 10. Empirical variogram for Tanzania. SI Section A4. Parameter estimation and spatial prediction. SI Fig. 11. Kenya’s triangulated mesh to build the SPDE model. SI Fig. 12. Uganda’s triangulated mesh to build the SPDE model. SI Fig. 13. Tanzania’s triangulated mesh to build SPDE model. SI Section A5. Exceedance probabilities. SI Fig. 14. Exceedance probability for Kenya, Uganda, and mainland Tanzania. SI Section A6. Validating the assumed spatial correlation function. SI Fig. 15. Empirical variogram estimated from the mixed effect model, including the 95% confidence interval band obtained from a simulation from the fitted model in Kenya. SI Fig. 16. Empirical variogram estimated from the mixed effect model, including the 95% confidence interval band obtained from a simulation from the fitted model in Uganda. SI Fig. 17. Empirical variogram estimated from the mixed effect model, including the 95% tolerance band obtained from a simulation from the fitted model in Tanzania. Fig. 18. The absolute number of women with less than 4 ANC visits across health planning units in Uganda, [file 12884_2022_5238_MOESM1_ESM.docx]

# Spatial inequities in antenatal care in East Africa: Additional File 1

# Section A1: Key indicators for Kenya, Uganda, and mainland Tanzania

SI Figure 1: health planning units in Uganda, Kenya, and Mainland Tanzania (see footnote)


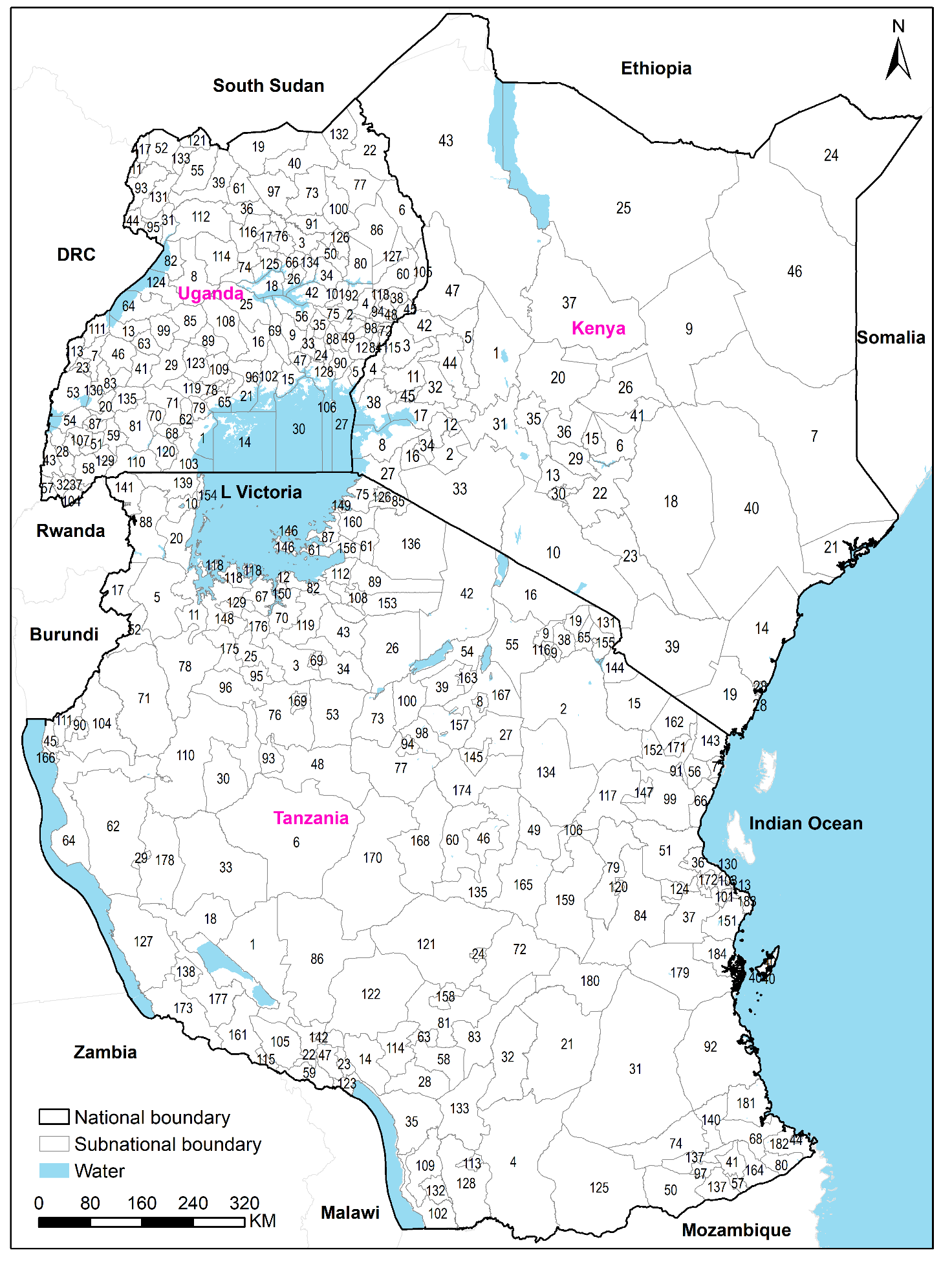


**SI Figure 1 footnote.**

**Kenya counties**: Baringo (1), Bomet (2), Bungoma (3), Busia (4), Elgeyo-Marakwet (5), Embu (6), Garissa (7), Homa Bay (8), Isiolo (9), Kajiado (10), Kakamega (11), Kericho (12), Kiambu (13), Kilifi (14), Kirinyaga (15),Kisii (16), Kisumu (17), Kitui (18), Kwale (19), Laikipia (20), Lamu (21), Machakos (22), Makueni (23), Mandera (24), Marsabit (25), Meru (26), Migori (27), Mombasa (28), Murang'a (29), Nairobi (30), Nakuru (31), Nandi (32), Narok (33), Nyamira (34), Nyandarua (35), Nyeri (36), Samburu (37), Siaya (38), Taita Taveta (39), Tana River (40), Tharaka-Nithi (41), Trans Nzoia (42), Turkana (43), Uasin Gishu (44), Vihiga (45), Wajir (46), West Pokot (47)

**Uganda districts** : Masaka (1), Butebo (2), Alebtong (3), Bukedea (4), Busia (5), Moroto (6), Kabarole (7), Masindi (8), Kamuli (9), Budaka (10), Maracha (11), Tororo (12), Kagadi (13), Kalangala (14), Buikwe (15), Luwero (16), Kole (17), Amolatar (18), Lamwo (19), Ibanda (20), Wakiso (21), Kaabong (22), Bunyangabu (23), Iganga (24), Nakasongola (25), Kaberamaido (26), Namayingo (27), Rukungiri (28), Mubende (29), Buvuma (30), Pakwach (31), Rubanda (32), Luuka (33), Soroti (34), Kaliro (35), Omoro (36), Rukiga (37), Kween (38), Amuru (39), Kitgum (40), Kyegegwa (41), Serere (42), Kanungu (43), Zombo (44), Bukwo (45), Kyenjojo (46), Jinja (47), Kapchorwa (48), Butaleja (49), Amuria (50), Sheema (51), Yumbe (52), Kasese (53), Rubirizi (54), Adjumani (55), Buyende (56), Kisoro (57), Ntungamo (58), Mbarara (59), Nakapiripirit (60), Gulu (61), Bukomansimbi (62), Kibaale (63), Kikuube (64), Mpigi (65), Dokolo (66), Mitooma (67), Lwengo (68), Kayunga (69), Lyantonde (70), Ssembabule (71), Bududa (72), Agago (73), Apac (74), Pallisa (75), Lira (76), Kotido (77), Butambala (78), Kalungu (79), Katakwi (80), Kiruhura (81), Buliisa (82), Kamwenge (83), Manafwa (84), Kyankwanzi (85), Napak (86), Buhweju (87), Namutumba (88), Kiboga (89), Bugiri (90), Otuke (91), Kumi (92), Arua (93), Sironko (94), Nebbi (95), Kampala (96), Pader (97), Mbale (98), Kakumiro (99), Abim (100), Ngora (101), Mukono (102), Kyotera (103), Kabale (104), Amudat (105),Mayuge (106), Bushenyi (107), Nakaseke (108), Mityana (109), Isingiro (110), Ntoroko (111), Nwoya (112), Bundibugyo (113), Kiryandongo (114), Namisindwa (115), Oyam (116), Koboko (117), Bulambuli (118), Gomba (119), Rakai (120), Moyo (121), Kibuku (122), Kassanda (123), Hoima (124), Kwania (125), Kapelebyong (126), Nabilatuk (127), Bugweri (128), Rwampara (129), Kitagwenda (130), Madi Okollo (131), Karenga (132), Obongi (133), Kalaki (134), Kazo (135)

**Mainland Tanzania councils**: Songwe (1), Simanjiro (2), Shinyanga Rural (3), Namtumbo (4), Biharamulo (5), Sikonge (6), Tanga (7), Babati Urban (8), Arusha (9), Bukoba Rural (10), Chato (11), Ilemela (12), Temeke (13), Makete (14), Same (15), Longido (16), Ngara (17), Mpimbwe (18), Siha (19), Muleba (20), Ulanga (21), Mbeya Rural (22), Busokelo (23),Iringa Urban (24), Msalala (25), Meatu (26), Kondoa (27), Njombe Urban (28), Mpanda Urban (29), Urambo (30), Liwale (31), Malinyi (32), Mlele (33), Kishapu (34), Ludewa (35), Bagamoyo (36), Kisarawe (37), Meru (38), Mbulu (39), Mafia (40), Newala (41), Ngorongoro (42), Maswa (43), Mtwara Urban (44), Kigoma Rural (45), Dodoma Urban (46), Rungwe (47), Uyui (48), Kongwa (49), Nanyumbu (50), Chalinze (51), Kakonko (52), Igunga (53), Karatu (54), Monduli (55), Muheza (56), Newala TC (57), Njombe Rural (58), Ileje (59), Bahi (60), Bunda (61), Tanganyika (62), Makambako TC (63), Uvinza (64), Hai (65), Pangani (66), Sengerema (67), Mtama (68), Shinyanga Urban (69), Misungwi (70), Kibondo (71), Kilolo (72), Iramba (73), Nachingwea (74), Rorya (75), Nzega (76), Ikungi (77), Bukombe (78), Mvomero (79), Nanyamba (80), Mufindi (81), Magu (82), Kilombero (83), Morogoro (84), Tarime (85),Chunya (86), Musoma (87), Karagwe (88), Bariadi (89), Kasulu TC (90), Korogwe TC (91), Kilwa (92), Tabora MC (93), Singida Urban (94), Kahama TC (95),Ushetu (96), Masasi TC (97), Singida (98), Handeni (99),Mkalama (100), Ilala (101), Nyasa (102), Ubungo MC (103), Kasulu (104), Mbozi (105), Gairo (106), Moshi Municipal (107), Bariadi TC (108), Mbinga (109), Kaliua (110), Buhigwe (111), Busega (112), Songea Urban (113), Wanging'ombe (114), Tunduma (115), Arusha Urban (116), Kilindi (117), Buchosa (118), Kwimba (119), Morogoro Urban (120), Iringa Rural (121), Mbarali (122), Kyela (123), Kibaha (124), Tunduru (125), Tarime TC (126), Nkasi (127), Songea Rural (128), Geita TC (129), Kinondoni (130), Rombo (131), Mbinga TC (132), Madaba (133), Kiteto (134), Chamwino (135), Serengeti (136), Masasi (137), Sumbawanga MC (138), Missenyi (139), Ruangwa (140), Kyerwa (141), Mbeya MC (142), Mkinga (143), Mwanga (144), Kondoa (145), Ukerewe (146), Handeni TC (147), Geita (148), Musoma Municipal (149), Nyamagana (150), Mkuranga (151), Korogwe (152), Itilima (153), Bukoba Urban (154), Moshi (155), Bunda TC (156), Hanang (157), Mafinga TC (158), Kilosa (159), Butiama (160), Momba (161), Lushoto (162), Mbulu TC (163), Tandahimba (164), Mpwapwa (165), Kigoma MC-Ujiji (166), Babati (167), Manyoni (168), Nzega TC (169), Itigi (170), Bumbuli (171), Kibaha Urban (172), Kalambo (173), Chemba (174), Mbogwe (175), Nyang'wale (176), Sumbawanga DC (177), Nsimbo (178), Rufiji (179), Ifakara (180), Lindi Urban (181), Mtwara Rural (182), Kigamboni (183), Kibiti (184).

**SI Table 1: Key indicators for Kenya, Uganda, and Tanzania**

| Indicator | Kenya | Uganda | Tanzania | Source |
| --- | --- | --- | --- | --- |
| Total population | 48.7 million | 41.6 million | 55.9 million | (1–3)* |
| Intercensal growth rate | 2.2% | 3.1% | 3.1% | (1,3,4) |
| Total fertility rate, | 3.4 | 4.8 | 4.9 | (5)^b^ |
| Crude Birth Rate (per 1000 people) | 28 | 37 | 36 | (5)^b^ |
| Number of births | 1,506,000 | 1,670,000 | 2,153,000 | (6)* |
| Minimum number of antenatal visits | 4 | 4 | 4 | (7,8) |
| Number of districts (Figure 1) | 47 counties | 135 districts | 184 councils | (3,4,9) |
| Percentage of population living below poverty line | 36.1^d^ | 20.3^c^ | 26.4^a^ | (5) |
| Female literacy rates (aged 15+) | 78^c^ | 71^c^ | 73^d^ | (5) |
| Number of health facilities | 13,657** | 6,937** | 8,458* | (1,10,11) |
| Percentage of population within 5km of health facility | 91% | 86% | 85% | (7,12,13) |
| Maternal mortality ratio (per 100, 000 live births) | 342 | 375 | 524 | (14)^a^ |
| Stillbirth rate per 1,000 total births | 19.8 | 17.8 | 18.8 | (15)^b^ |

Years: d 2015, a 2017, 2018^c^ b 2019, *2020, **2022

# Section A2: Sampling in the malaria indicator surveys.

Each malaria indicator survey (MIS) followed a two-stage sample design The first stage involved selecting sample points (clusters) consisting of enumeration areas (EAs) from a national frame. The clusters were selected with probability proportional to EA population size and with independent selection in each sampling stratum. The second stage involved a random selection of households per sampled cluster from a list of all households. The surveys were designed to allow estimates for the entire country, urban and rural areas, and regions (5 in Kenya, 15 in Uganda and 31 in Tanzania).

In Kenya, 8,845 households (30 per cluster) were selected from 301 sampled clusters (134 urban and 167 rural). High to moderate risk malaria zones were oversampled, and the sample distributed over all the 47 counties. Tanzania had 442 clusters (127 urban and 315 rural) with 22 households randomly selected from per cluster resulting to 9,724 households. In Uganda, 320 clusters were selected (84 urban and 236 rural) with an additional 22 clusters from a refugee sampling frame. From each sampled cluster, 28 households were selected resulting to 8,878 households. Urban areas were oversampled to produce robust estimates for urban areas.

A small percentage of the sampled women who had missing responses for variables of interest were excluded from the analysis. The distribution of proportions of missing observations for each variable across countries are shown in Table 2 below.

**SI Table 2: Proportions of missing observations across variables of interest extracted from MIS survey for Kenya, Uganda, and mainland Tanzania**

| Variable | Kenya n (%) | Uganda n (%) | mainland Tanzania n (%) |
| --- | --- | --- | --- |
| ANC4+ * | 28 (1.4) | 36 (0.9) | 93 (2.1) |
| ANC initiation for women with at least ≥ 1 visit * | 23 (1.2) | 39 (0.9) | Not collected |
| Decision to seek ANC services * | 17 (0.9) | Not collected | |
| Maternal education | No missing values | | |
| Household wealth |  |  |  |
| Birth order |  |  |  |
| Health media exposure |  |  |  |
| Travel time to the nearest public health facility |  |  |  |

*Missing values were computed among mothers with at least ≥ 1 ANC visit

# S1 Section A3: Exploration the relationship between the prevalence and covariates.

Exploratory analysis is the first stage of any geostatistical analysis (16). It entails visualizing the distribution of sampled locations (Figure 1A in manuscript), examining the correlation between covariates, assessment of the relationship between ANC4+ and covariates, and testing for residual spatial correlation (16). Specifically, correlation among predictors was checked by through Pearson’s correlation test with the corrplot package in R (17). The pair of predictors with Pearson correlation greater than 0.7 were regarded as highly correlated (SI Figures 2 to 4). The association between the predictors and women that had at least four antenatal visits (ANC4+) was examined through the empirical logit (18) of ANC4+. We also used scatter plots to visually examine the association between the covariates and the empirical logit prevalence of women that had ANC4+ visits during pregnancy (SI Figures 5-7).

To select the best model, we fit a non-spatial (generalized linear) model relating the predictors with the prevalence. We used forward and backward stepwise selection to select a parsimonious set of predictors required for the prediction of the coverage among all the candidates set of predictors. The final set of predictors results in a model with the lowest Akaike information criterion (AIC). In the final stage of explanatory analysis, we assessed the evidence of residual spatial variation. This is the variation that is not captured by the covariates retained in the previous stage. We used an empirical variogram to test whether this residual variation exhibited spatial correlation.

S1 Figure 2: Correlation plot for Kenya


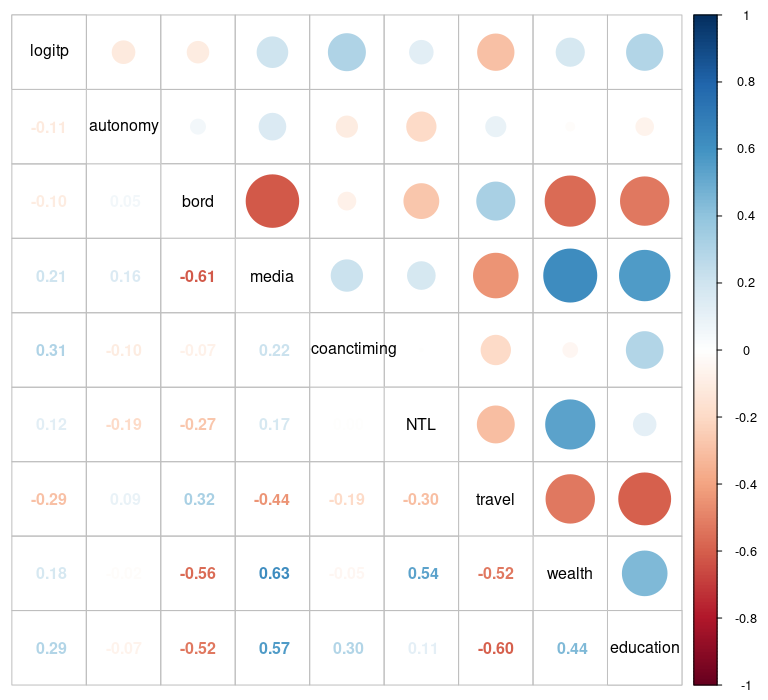


SI Figure 3:correlation plot for Tanzania


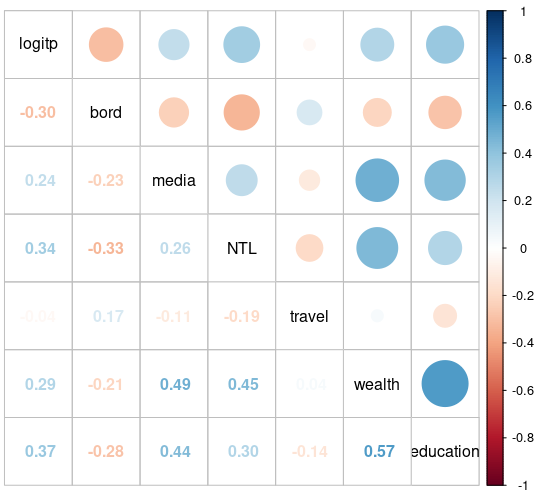


SI Figure 4: Correlation plot for Uganda


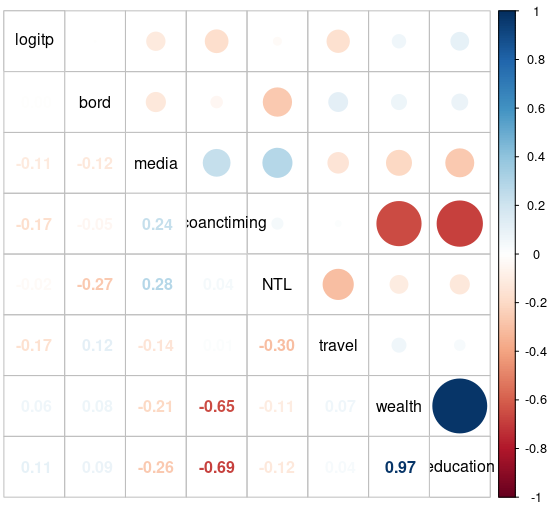


## Scatter plots

S1 Figure 5:Relationship between the empirical coverage of ANC4+ and the predictors (Decision to seek ANC services – (maternal autonomy), birth order, Night-time lights (NTL), ANC timing and travel time) for Kenya.


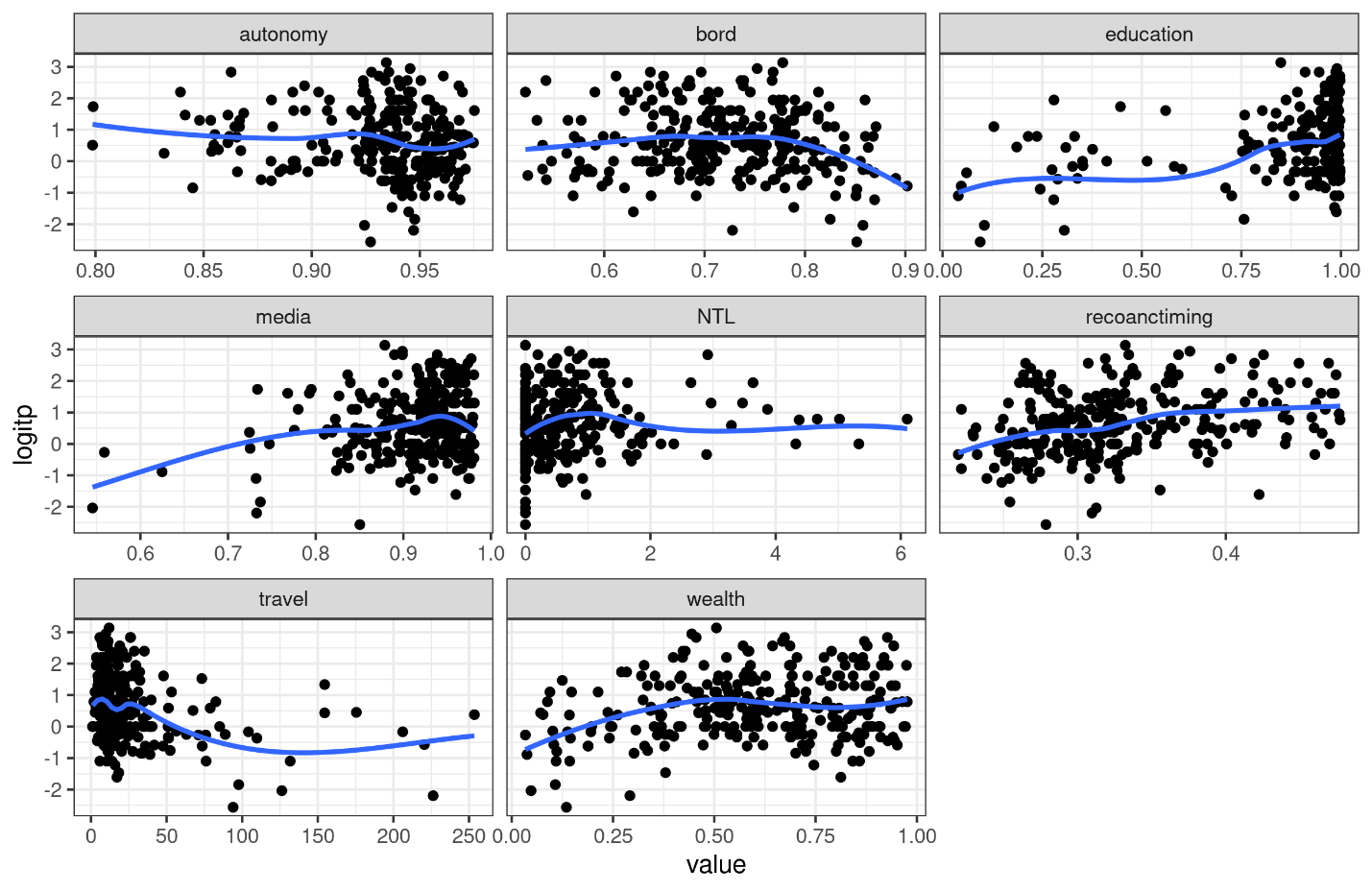


S1 Figure 6: Relationship between the empirical coverage of ANC4+ and the predictors (birth order, Night-time lights (NTL) and travel time) for Tanzania


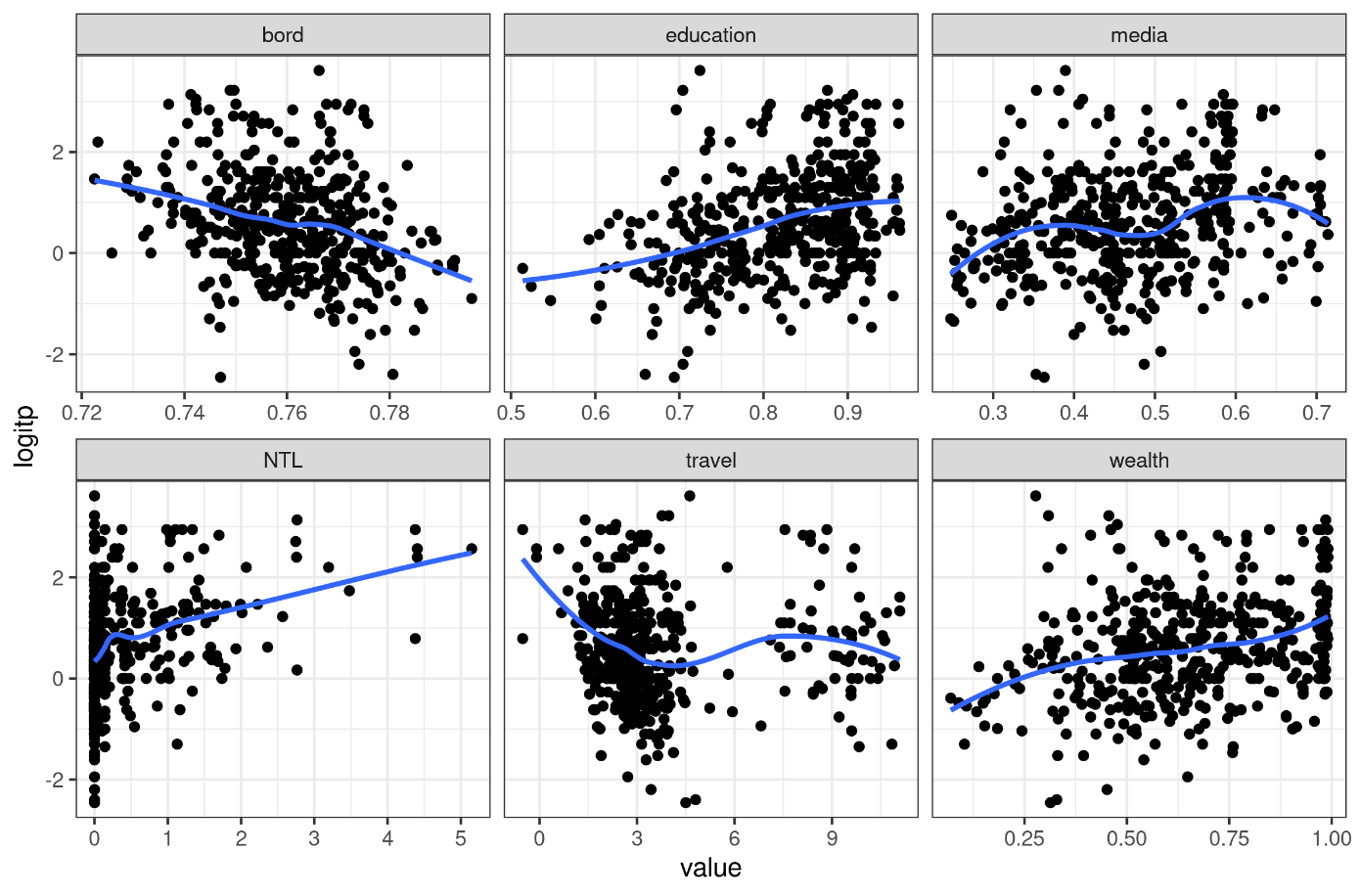


SI Figure 7:Rrelationship between the empirical prevalence of ANC4+ and the predictors (Night-time lights (NTL), ANC timing and travel time) for Uganda.


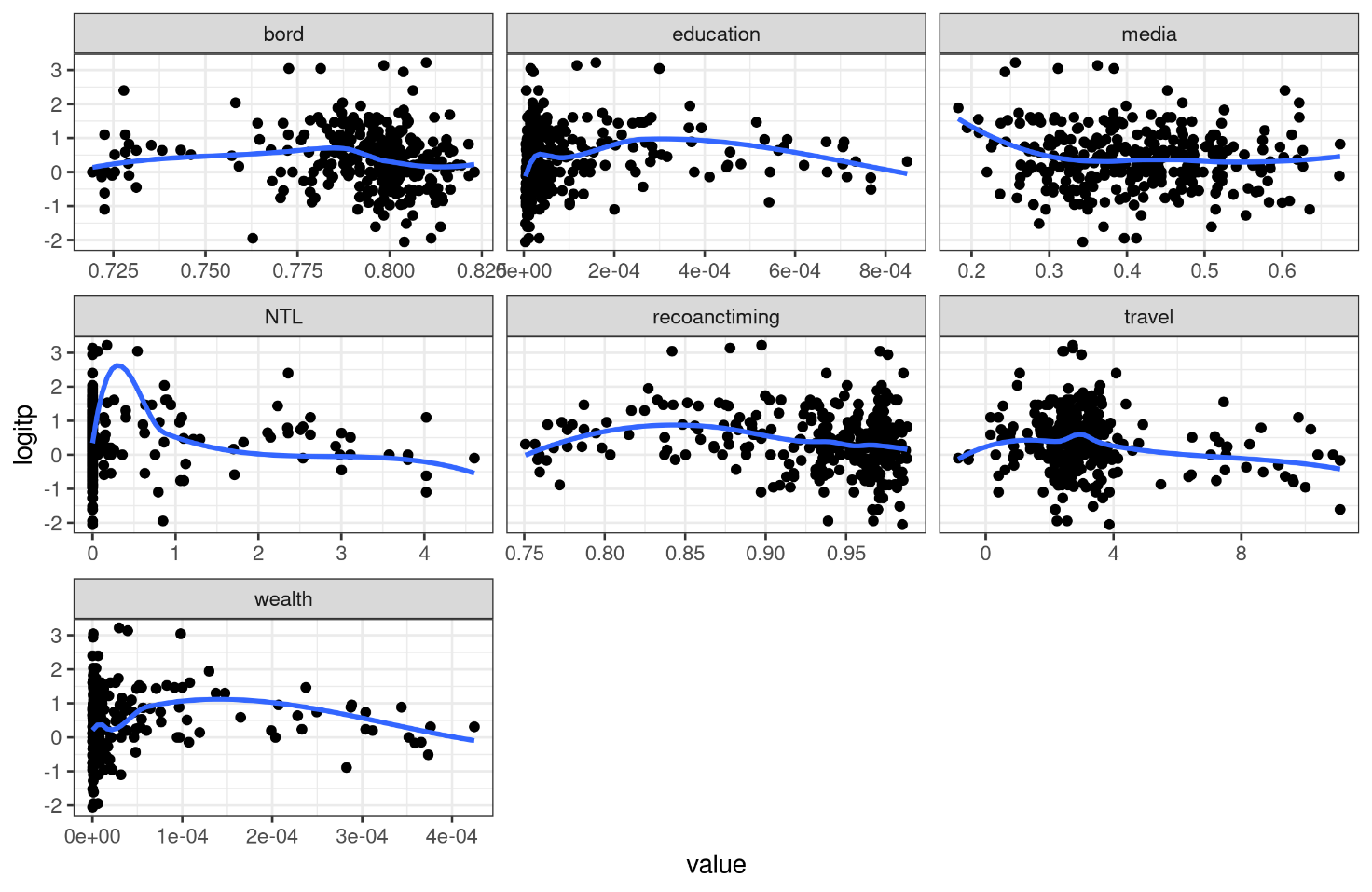


## Testing for residual spatial variation

The empirical variogram is used to test for the presence of residual spatial correlation after fitting a non-spatial model to the data. Empirical variogram describes the spatial dependence in the data and estimates the autocorrelation structure of the underlying stochastic process. We used the Monte-Carlo strategy to construct 95% confidence interval band to establish whether the observed patterns are or are not compatible with random fluctuations.

SI Figure 8: Empirical variogram including the 95% confidence interval obtained from a Monte Carlo strategy under the null hypothesis that there is no spatial correlation (blue-shaded area) in Kenya.


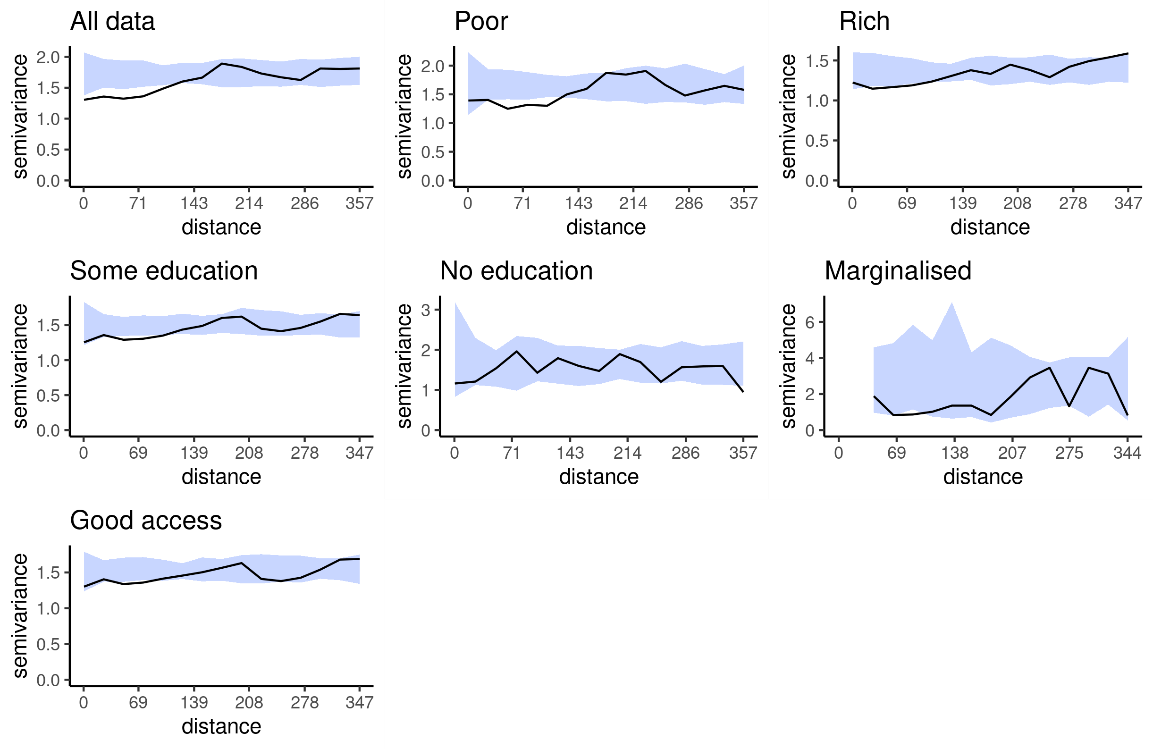


SI Figure 9: Empirical variogram including the 95% confidence interval obtained from a Monte Carlo strategy under the null hypothesis that there is no spatial correlation (blue-shaded area) in Uganda.


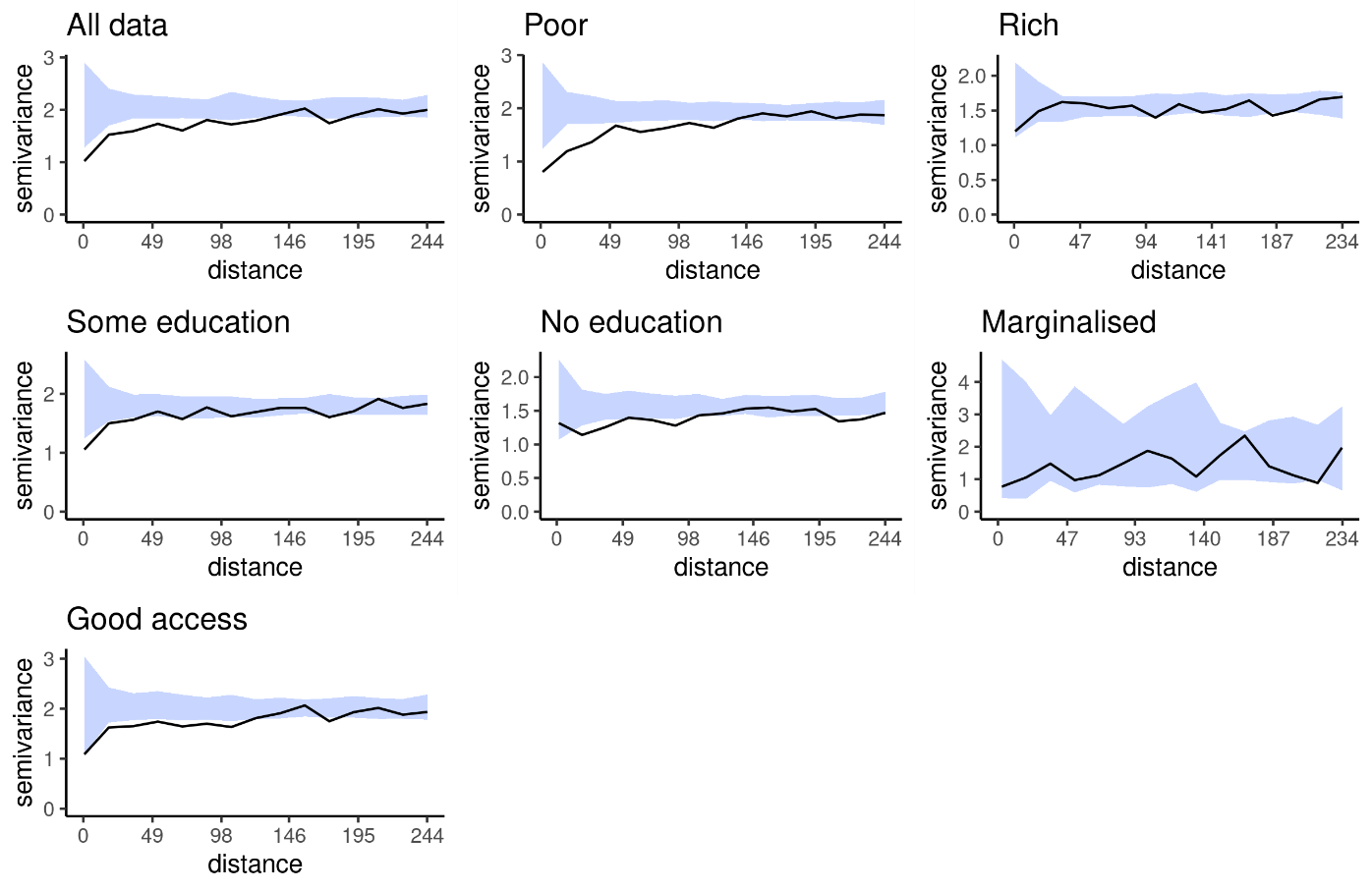


SI Figure 10: Empirical variogram including the 95% confidence interval obtained from a Monte Carlo strategy under the null hypothesis that there is no spatial correlation (blue-shaded area) in Tanzania.


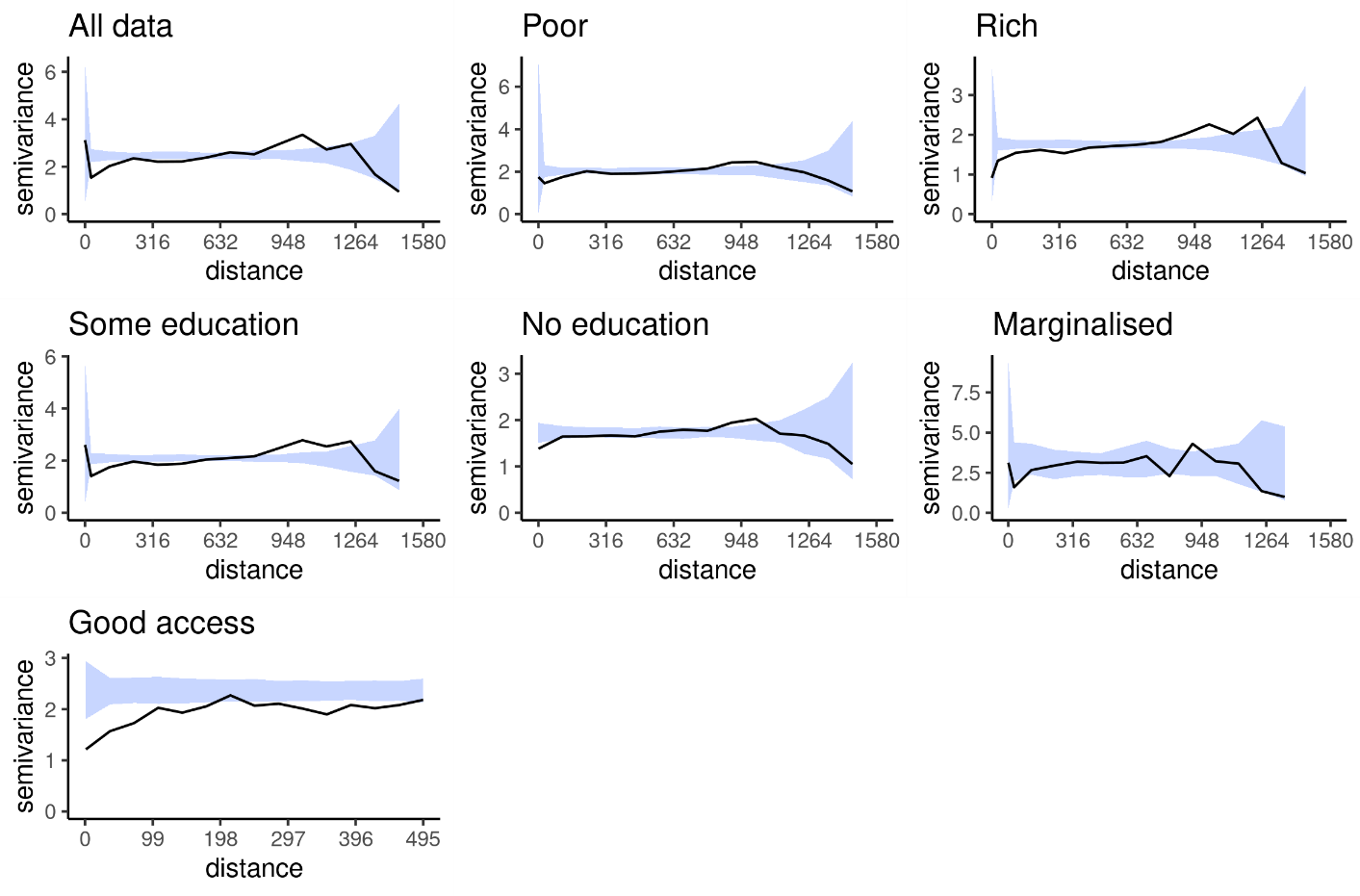


# SI Section A4: Parameter estimation and spatial prediction

We fit a geostatistical model for each country and strata. We modelled the number of women that attended ANC more than 4 times $Y_{i}$ among the total number of surveyed women ($m_{i}$) at location x as a binomial random variable. Binomial geostatistical model can be written hierarchically as:

$$Y_{i}/S\left( x \right),Z\sim Binomial\left( P_{i},m_{i} \right)$$

$$Logit\left( P_{i} \right)=d^{'}\beta+S\left( x \right)+Z$$

$$S\left( x \right)/\sigma^{2},\phi\sim GP\left( 0,\Sigma\left( \sigma^{2},\phi\right) \right)$$

$$Z\sim N\left( 0,\tau^{2} \right)$$

The logit-transformed prevalence of ($P_{i}$) was specified as a linear combination of the predictors (d), spatial random effects (S(x)) and non-spatial random effects (Z). The regression coefficient is denoted as 𝜷. The spatial covariance, 𝛴, is modelled using an isotropic and stationary Matérn function. The random effects were used to capture the unexplained variation, where S(x) captures the variation between locations and Z captures the variation within the locations, while the fixed effects (predictors) were used to capture the explained variation.

The Matérn covariance function used has three parameters namely, variance ($\sigma^{2}$), scale or range ($\phi$) and smoothness parameter ($\kappa$). The smoothness parameter was fixed at 1.5 which corresponds to using an exponential covariance function. The fixed effects were assigned independent vague Gaussian priors with mean zero and precision equal to 0.001. The parameters of the covariance function were assigned a Penalized Complexity priors (19). The model was fitted in R-INLA (20) using the stochastic partial differential equations (SPDE) approach to approximate the continuous spatiotemporal Gaussian random fields (S(x)). We constructed triangulated mesh for the SPDE approximation using a simplified polygon boundary. The triangulated mesh for the geostatistical models used for the three countries is shown in SI Figures 11- 13.

SI Figure 11: Kenya’s triangulated mesh to build the SPDE model.


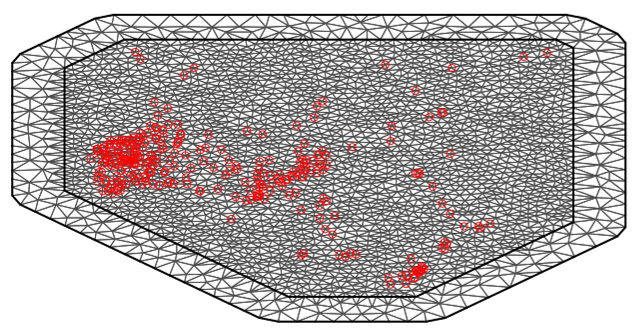


SI Figure 12: Uganda’s triangulated mesh to build the SPDE model.


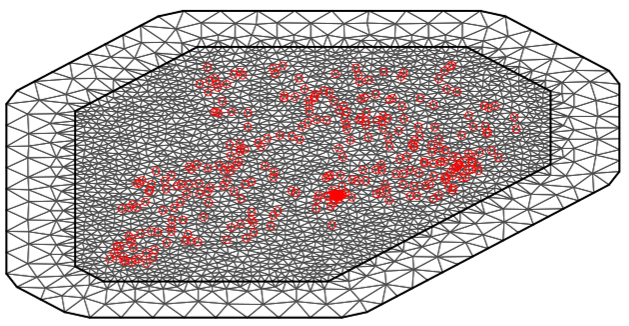


SI Figure 13:Tanzania’s triangulated mesh to build SPDE model


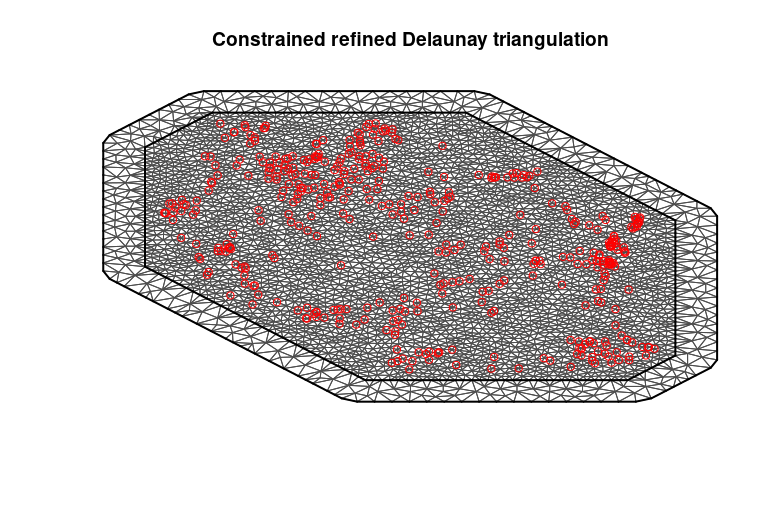


# SI Section A5: Exceedance probabilities

We assessed the likelihood of an area having ANC4+ coverage above 70% (target coverage) per district based on the EPMM strategy that aims for at least 80% of all subnational units within a country to have over 70% of the pregnant women having ANC4+ by 2025 (21). Formally, the fitted spatial model was used to compute the EP that an area has ANC4+ above 70% as expressed in Equation 2 at 3km spatial resolution. $EP_{x}$ is the exceedance probability at location x, $P_{x}$ is the ANC4+ coverage in location x and l is 70%. An EP value close to 100% indicates that ANC4+ is highly likely to be above the target coverage l; if close to 0%, ANC4+, is highly likely to be below the target coverage l; if close to 50%, ANC4+, is equally likely to be above or below the target coverage l, the highest level of uncertainty. The results are shown in SI Figure 14.

$EP_{x}=\Pr ob\left(P_{x}>l|data\right)$ --------- Equation 2

**SI Figure 14: Exceedance probability for a 70% ANC4+ target coverage with a likelihood of ≥90% (light green) or <10% (orange) disaggregated by household wealth quintile (A, D), education (B, E) and travel time to the nearest facility (C, F) in Kenya, Uganda, and mainland Tanzania.**


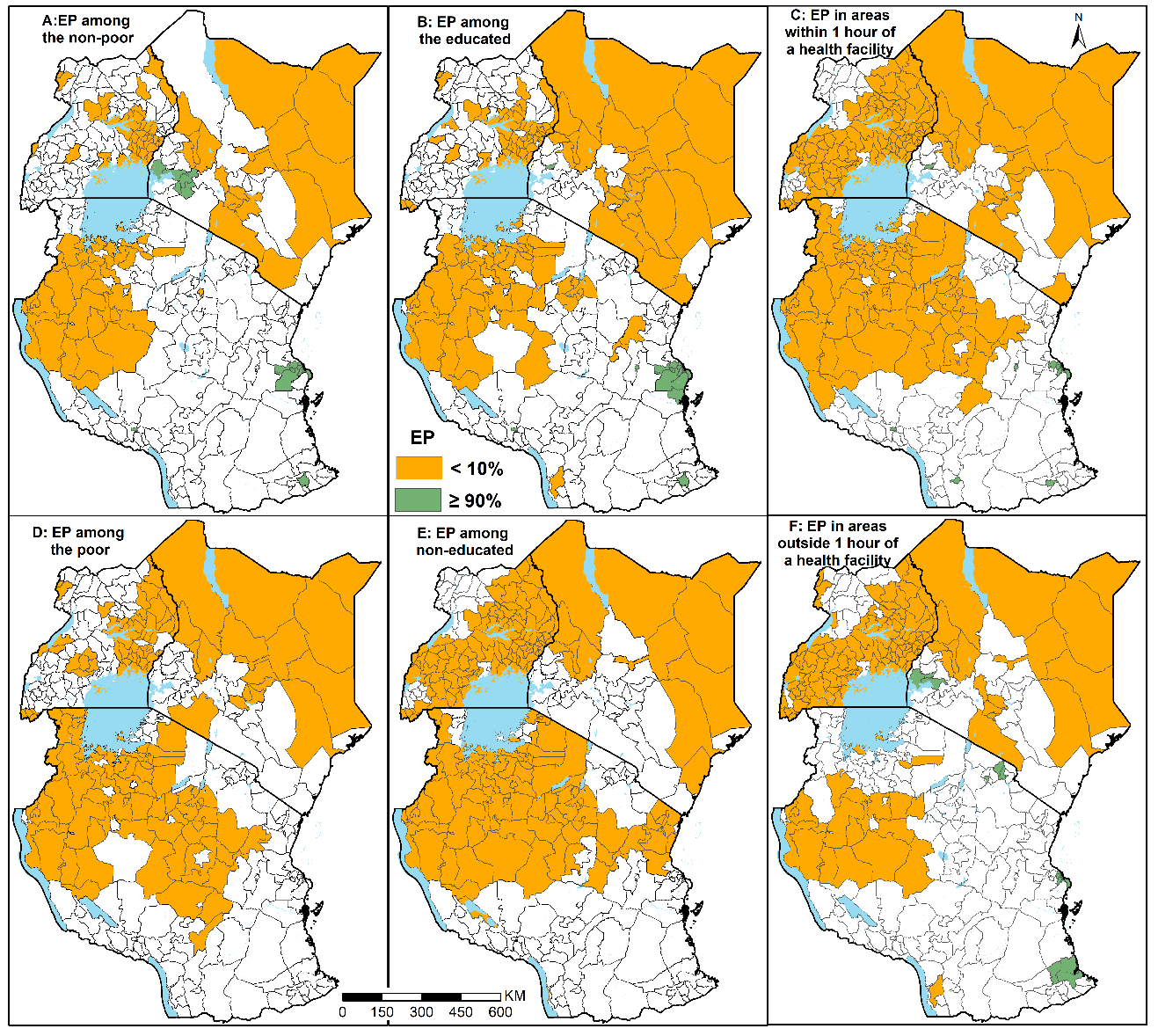


# SI Section A6: Validating the assumed spatial correlation function.

The procedure for validating the assumed spatial correlation function is as follows:

1. Fit to the data a generalised binomial linear mixed model and estimate the random effect W such that W is independent and identically distributed normal variables.
2. calculate the empirical variogram V_0_ based on W.
3. fix the parameters of the model and simulate k=1000 Gaussian process S(x) and Gaussian noise Z at each of the observed locations x= i=1,...n;
4. compute the W_i_ = S(x) + Z_i_ ,
5. compute the variogram, V_k_ based on W_i_ ,
6. compute the 95% tolerance interval of V_k_

If the variogram V_0_ lies within the resulting 95% confidence interval band, it means that true covariance function for S(x) exactly corresponds to the one adopted for the analysis. The results for Kenya, Tanzania and Uganda are shown in SI Figures 15 – 17. The results show that the adopted covariance function is compatible with the intrinsic spatial correlation in the data.

SI Figure 15: Empirical variogram estimated from the mixed effect model, including the 95% confidence interval band obtained from a simulation from the fitted model (blue-shaded area) in Kenya.


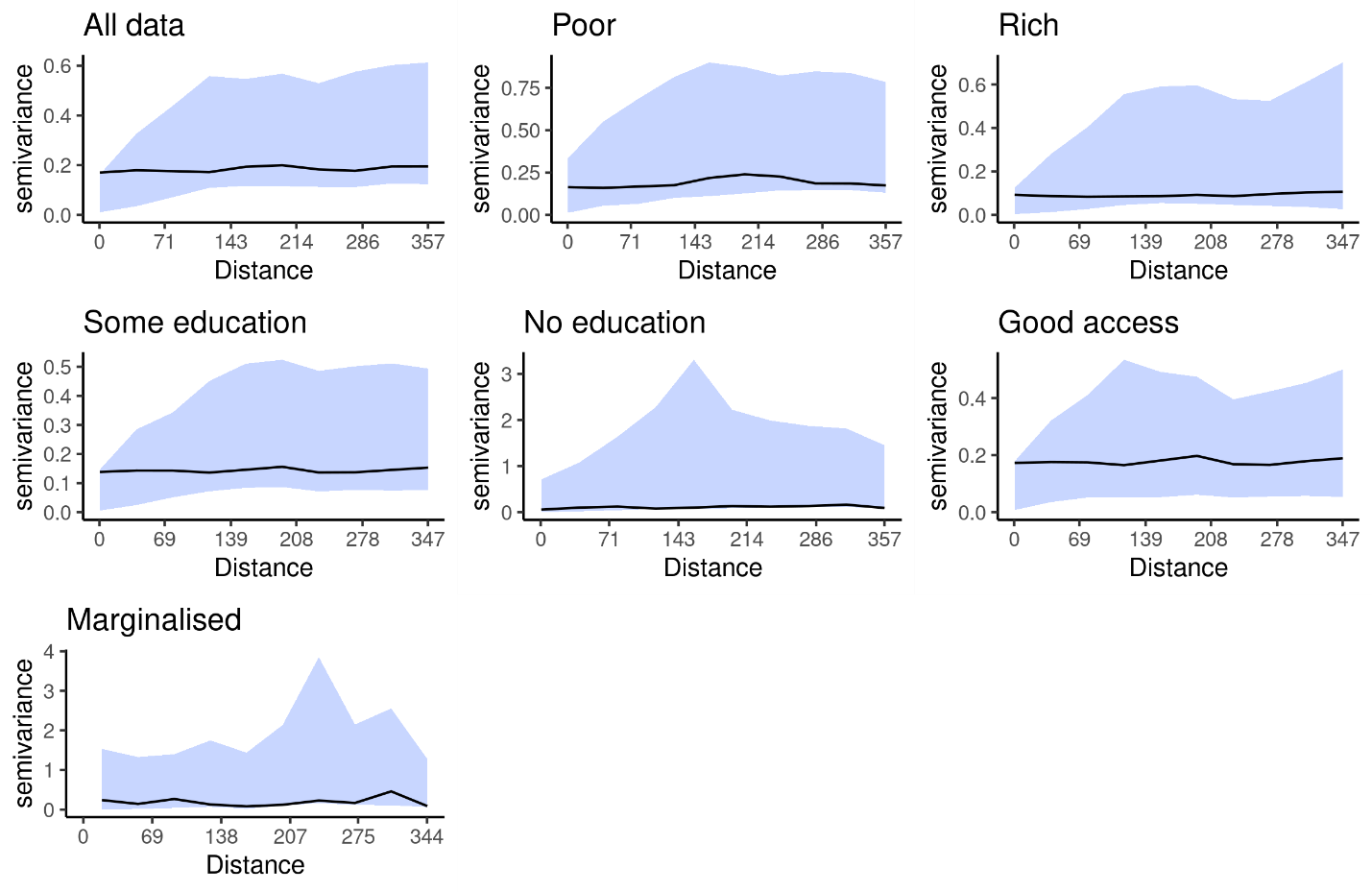


SI Figure 16: Empirical variogram estimated from the mixed effect model, including the 95% confidence interval band obtained from a simulation from the fitted model (blue-shaded area) in Uganda.


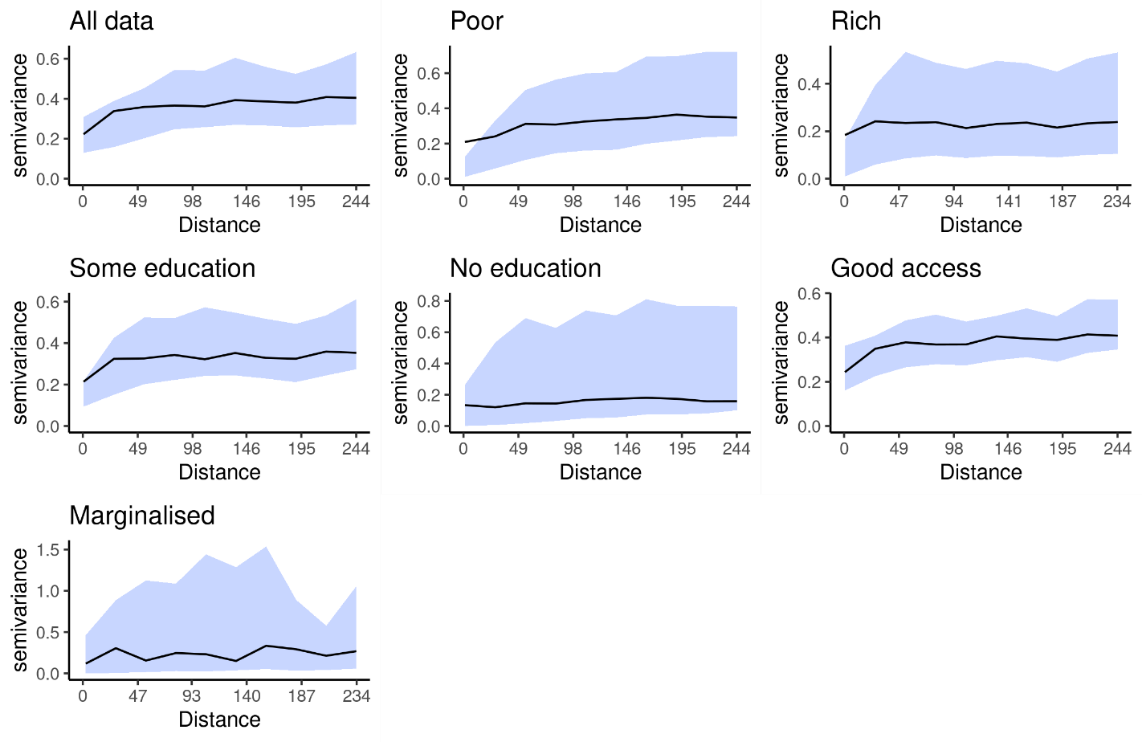


SI Figure 17: Empirical variogram estimated from the mixed effect model, including the 95% tolerance band obtained from a simulation from the fitted model (blue-shaded area) in Tanzania.


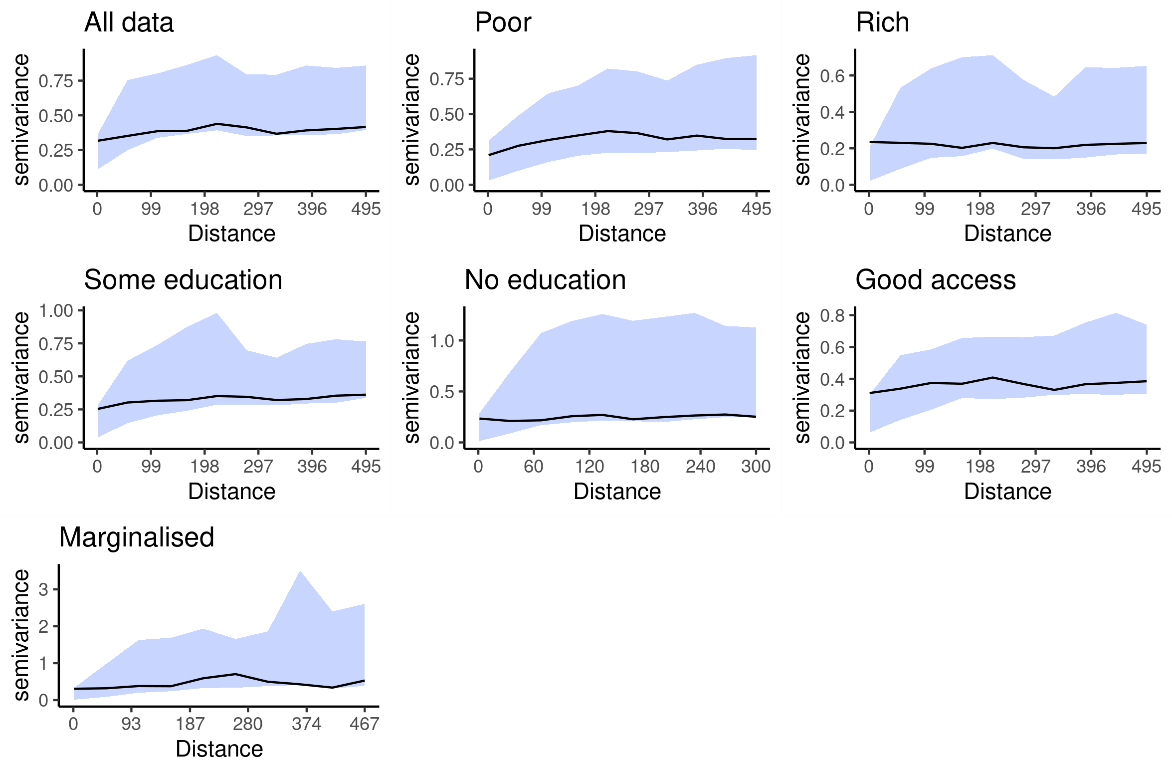


Figure 18: The absolute number of women with less than 4 ANC visits across health planning units in Uganda, Kenya, and mainland Tanzania


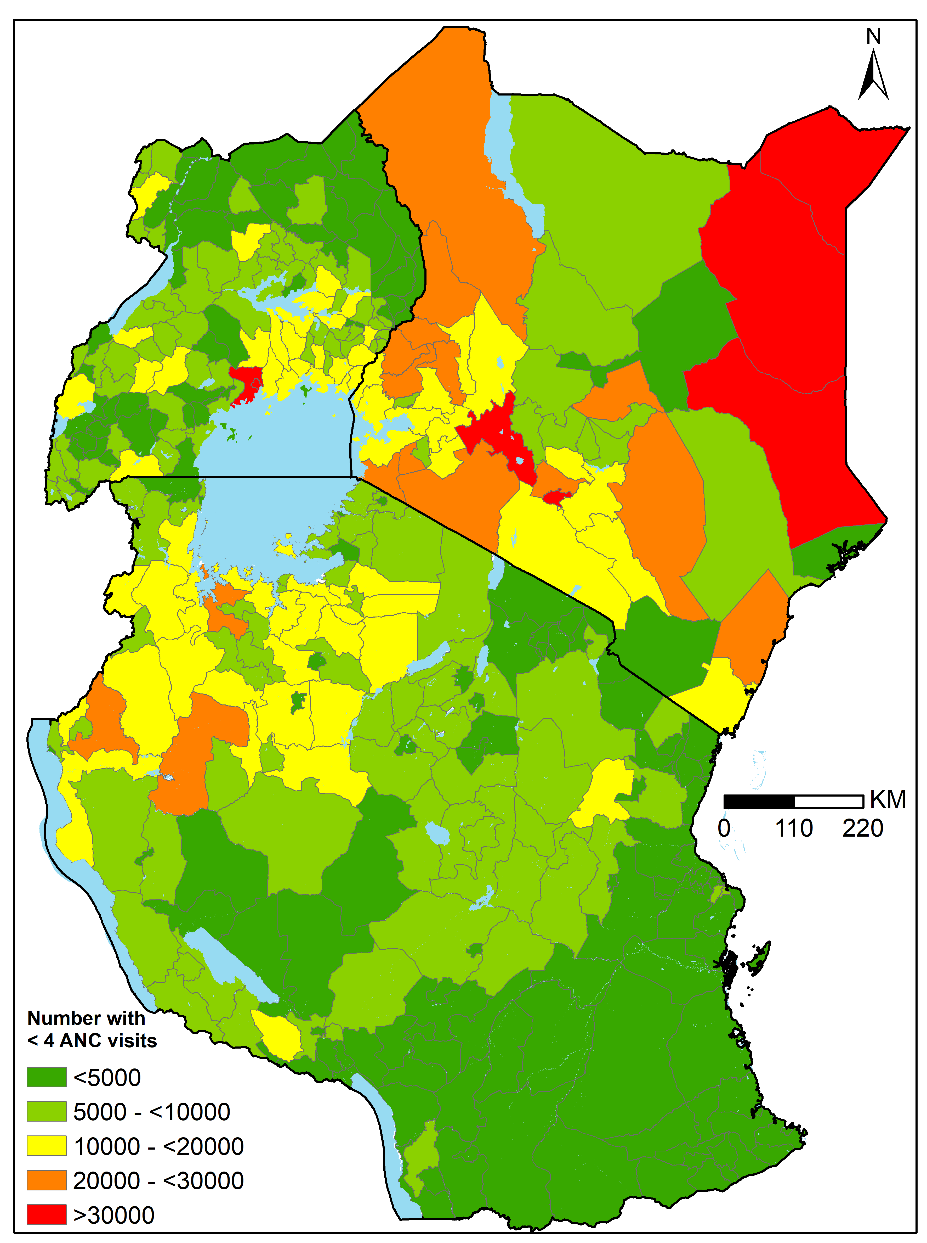


# SI section A8: Parameter estimates and corresponding 95% credible interval

| **Kenya** **Estimate (95% CI)** | | | | | | | |
| --- | --- | --- | --- | --- | --- | --- | --- |
|  | **Before stratification** | **Poor** | **Non-poor** | **No education** | **Some education** | **Marginalised** | **Good accessibility** |
| Intercept | -3.165 (-5.294, -0.947) | 1.975 (-3.150, 7.429) | -0.147 (-0.901, 0.594) | -3.284 (-7.944, 1.922) | -1.326 (-2.123, -0.555) | -3.622 (-12.358, 5.288) | -1.495 (-2.392, -0.615) |
| Decision to seek ANC services |  | -3.914 (-9.571, 1.390) |  |  |  | -5.288 (-14.127, 3.367) |  |
| High order birth | 1.101 (-1.126, 3.222) |  |  |  | -1.327 (-3.672, 0.889) |  |  |
| Health media exposure |  |  |  | 1.617 (-2.725, 5.324) |  | 3.954 (0.416, 7.609) |  |
| ANC initiation for women with at ≥ 1 visit | 5.603 (2.964, 8.311) | 3.797 (0.078, 7.599) | 2.051 (0.785, 3.311) | 7.096 (-3.835, 17.672) | 7.620 (3.253, 8.145) | 16.764 (6.713, 27.467) | 4.861 (2.320, 7.474) |
| Nighttime light lights | 0.098 (-0.057, 0.256) | 0.388 (-0.057, 0.860) |  |  | 0.166 (0.036, 0.301) |  | 0.132 (0.003, 0.266) |
| Travel time to nearest health facility |  |  |  | -0.005 (-0.010, -0.001) |  |  |  |
| Household Wealth | 0.531 (-0.138, 1.202) |  |  |  |  |  |  |
| Maternal education | 0.848 (0.164, 1.527) | 0.855 (0.191, 1.544) |  |  |  |  | 0.503 (-0.203, 1.208) |
| Signal variance | 0.341 (0.186, 0.54) | 0.49 (0.289, 0.743) | 0.165 (0.029, 0.418) | 0.557 (0.221, 1.03) | 0.282 (0.124, 0.498) | 0.335 (0.064, 0.826) | 0.328 (0.16, 0.552) |
| Range parameter | 67.80 (16.914, 198.72) | 63.39 (9.482, 232.00) | 187.00 (25.518, 814.00) | 184.00 (13.510, 883.47) | 66.50 (15.842, 206.00) | 283.00 (21.925, 1580.00) | 57.20 (14.56, 174.00) |
| Cluster precision | 1.77e+04 (1171.723, 62558.83) | 19970.43 (1480.233, 7.24e+04) | 1.88e+04 (1296.101, 6.75e+04) | 1.97e+04 (1400.101, 72760.23) | 1.85e+04 (1255.409, 6.66e+04) | 1.82e+04 (1235.316, 6.61e+04) | 1.82e+04 (1253.39, 6.52e+04) |

| **mainland Tanzania Estimate (95% CI)** | | | | | | | |
| --- | --- | --- | --- | --- | --- | --- | --- |
|  | **Before stratification** | **Poor** | **Non-poor** | **No education** | **Some education** | **Marginalised** | **Good accessibility** |
| Intercept | 5.168 (-0.239, 10.574) | 6.231 (-1.628, 14.167) | 4.768 (-1.625, 11.102) | 4.304 (-5.800, 14.478) | 8.030 (2.494, 13.575) | -3.216 (-6.069, -0.534) | 7.321 (1.376, 13.265) |
| Birth order | -9.877 (-16.573, -3.218) | -11.290 (-21.308, -1.437) | -7.398 (-15.288, -0.504) | -6.882 (-20.041, 6.139) | -10.568 (-17.698, -3.475) |  | -11.637 (-19.055, -4.267) |
| Health media exposure | -0.077 (-1.898, 1.692) |  |  | 0.844 (-1.965, 3.509) | 0.237 (-1.759, 2.184) | -0.559 (-5.177, 3.683) |  |
| Nighttime light lights | 0.303 (0.127, 0.482) | 0.345 (-0.409, 1.129) | 0.250 (0.074, 0.427) |  | 0.299 (0.100, 0.501) | 0.405 (-0.086, 0.899) | 0.154 (-0.042, 0.353) |
| Travel time to nearest health facility |  | -0.006 (-0.015, 0.002) |  |  |  |  | -0.390 (-0.619, -0.163) |
| Household Wealth |  |  |  | 1.082 (-0.097, 2.320) | 0.835 (0.025, 1.674) |  |  |
| Maternal Education | 3.568 (1.895, 5.271) | 3.325 (1.385, 5.286) | 1.879 (0.004, 3.752) |  |  | 5.107 (1.064, 9.549) | 2.487 (0.808, 4.172) |
| Signal variance | 0.586 (0.455, 0.727) | 0.662 (0.477, 0.861) | 0.459 (0.312, 0.636) | 0.759 (0.534, 1.04) | 0.614 (0.438, 0.786) | 0.94 (0.63, 1.33) | 0.528 (0.384, 0.686) |
| Range parameter | 168.355 (93.235, 282.680) | 207.00 (95.126, 407.00) | 209.00 (74.180, 448.00) | 138.00 (46.763, 322.44) | 247.758 (123.184, 500.00) | 350.00 (97.246, 846.00) | 206.269 (93.597, 394.728) |
| Cluster precision | 50.149 (23.879, 107.167) | 1.69e+04 (1198.411, 6.35e+04) | 1.76e+04 (1509.729, 5.87e+04) | 1.68e+04 (1118.619, 68583.15) | 5327.861 (206.945, 1.97e+04) | 7.38e+04 (1096.189, 4.86e+05) | 50.611 (20.531,  104.695) |

| **Uganda** **Estimate (95% CI)** | | | | | | | | |
| --- | --- | --- | --- | --- | --- | --- | --- | --- |
|  | **Before stratification** | **Poor** | **Non-poor** | **No education** | **Some education** | **Marginalised** | **Good accessibility** |  |
| Intercept | 5.729 (-3.479, 14.559) | 7.511 (-4.956, 19.581) | 6.694 (-2.958, 16.136) | 9.576 (-4.756, 23.778) | 4.761 (-4.206, 13.484) | 8.394 (-3.861, 20.569) | -0.514 (-19.556, 18.510) |  |
| Birth order | -5.720 (-15.028, 3.530) | -8.412 (-21.207, 4.233) | 16.136 (-16.751, 5.488) | -10.344 (-25.530, 4.590) | -4.955 (-14.664, 4.735) | -10.454 (-25.742, 4.891) | -5.207 (-14.709, 4.289) |  |
| Health media exposure |  |  |  | 0.432 (-1.605, 2.402) | -0.358 (-1.783, 1.095) |  |  |  |
| ANC in first trimester | -0.955 (-5.803, 4.605) | -0.623 (-7.171, 6.817) | -1.883 (-4.885, 1.486) | -1.572 (-9.562, 6.667) | -0.255 (-4.209, 4.314) |  | -0.360 (-7.139, 6.437) |  |
| Nighttime light lights | -0.133 (-0.339, 0.072) |  | -0.154 (-0.397, 0.088) |  | -0.145 (-0.366, 0.075) |  | -0.140 (-0.359, 0.079) |  |
| Travel time to nearest health facility | -0.198 (-0.387, -0.014) | -0.213 (-0.448, 0.011) | -0.263 (-0.561, 0.029) | -0.327 (-0.769, 0.053) | -0.185 (-0.388, 0.014) | -0.131 (-0.342, 0.075) | -0.227 (-62.311, 61.805) |  |
| Household Wealth | -0.399 (-62.423, 61.633) |  |  |  | -0.495 (-62.578, 61.536) |  |  |  |
| Education |  | -0.459 (-62.540, 61.569) |  |  |  |  |  |  |
| Signal variance | 0.613 (0.465, 0.797) | 0.698 (0.489, 0.955) | 0.486 (0.331, 0.674) | 0.611 (0.346, 0.996) | 0.559 (0.426, 0.726) | 0.51 (0.274, 0.859) | 0.53 (0.40, 0.69) |  |
| Range parameter | 123.00 (63.062, 226.00) | 169.00 (74.527, 339.00) | 43.90 (13.80, 99.70) | 465.00 (78.83, 1650.00) | 88.00 (45.58, 164.00) | 391.52 (35.125, 2050.00) | 177.148 (176.11, 177.82) |  |
| Cluster precision | 1.73e+04 (1188.291, 6.43e+04) | 2.15e+04 (2011.674, 7.83e+04) | 1.81e+04 (1266.144, 6.77e+04) | 1.88e+04 (1219.852, 6.67e+04) | 1.86e+04 (1296.167, 6.83e+04) | 19519.07 (1456.533, 6.91e+04) | 44.475 (44.394, 44.548) |  |

# References

1. Tanzania National Bureau of Statistics (TNBS). 2020 Tanzania in Figures [Internet]. 2021 [cited 2022 Feb 22]. Available from: https://www.nbs.go.tz/index.php/en/tanzania-in-figures/641-tanzania-in-figures-2020

2. Kenya National Bureau of Statistics. Kenya facts and figures [Internet]. 2021 [cited 2022 Feb 22]. Available from: https://www.knbs.or.ke/download/facts-figures-2021/?wpdmdl=6452&refresh=6214d19209dc21645531538

3. Uganda Bureau of Statistics. Uganda General Information [Internet]. 2022 [cited 2022 Feb 22]. Available from: https://www.ubos.org/uganda-profile/

4. The Kenya National Bureau of Statistics (KNBS). 2019 Population and Housing Census Reports [Internet]. 2020 [cited 2022 Feb 22]. Available from: https://www.knbs.or.ke/2019-kenya-population-and-housing-census-reports/

5. World Bank. Free and open access to global development data [Internet]. 2022 [cited 2022 Feb 22]. Available from: https://data.worldbank.org/

6. UNICEF. UNICEF Data Warehouse [Internet]. 2022 [cited 2022 Feb 22]. Available from: https://data.unicef.org/dv_index/

7. Republic of Uganda. Ministry of Health Strategic Plan 2020/21 - 2024/25. 2022 [Internet]. 2021 [cited 2022 Feb 22]. Available from: https://www.health.go.ug/cause/ministry-of-health-strategic-plan-2020-21-2024-25/

8. Minstry of Health and Sanitation and ministry of medical services. National Guidelines for Quality Obstetrics and Perinatal Care [Internet]. 2011 [cited 2022 Feb 22]. Available from: http://guidelines.health.go.ke/#/category/27/76/meta

9. Alegana VA, Macharia PM, Muchiri S, Mumo E, Oyugi E, Kamau A, et al. Plasmodium falciparum parasite prevalence in East Africa: Updating data for malaria stratification. PLOS Glob Public Health [Internet]. 2021;1(12)::e0000014. Available from: https://doi.org/10.1371/journal.pgph.0000014

10. Ministry of Health Republic of Uganda. Hospitals [Internet]. 2022 [cited 2022 Feb 22]. Available from: https://www.health.go.ug/hospitals/

11. Kenya Ministry of Health. Kenya Master health facility list. 2022. [Internet]. 2022 [cited 2022 Feb 22]. Available from: http://kmhfl.health.go.ke/#/facility_filter/results

12. Owino L, Wangong’u A, Were N, Maleche A. The missing link in Kenya’s universal health coverage experiment: a preventive and promotive approach to SRHR. Vol. 28, Sexual and Reproductive Health Matters. 2020.

13. Straneo M, Benova L, Hanson C, Fogliati P, Pembe AB, Smekens T, et al. Inequity in uptake of hospital-based childbirth care in rural Tanzania: Analysis of the 2015-16 Tanzania Demographic and Health Survey. Health Policy and Planning. 2021;36(9).

14. WHO. Trends in maternal mortality 2000 to 2017: estimates by WHO, UNICEF, UNFPA, World Bank Group and the United Nations Population Division [Internet]. Geneva: World Health Organization; 2019. Available from: https://apps.who.int/iris/handle/10665/327595

15. Hug L, You D, Blencowe H, Mishra A, Wang Z, Fix MJ, et al. Global, regional, and national estimates and trends in stillbirths from 2000 to 2019: a systematic assessment. The Lancet. 2021;398(10302).

16. Giorgi E, Fronterrè C, Macharia PM, Alegana VA, Snow RW, Diggle PJ. Model building and assessment of the impact of covariates for disease prevalence mapping in low-resource settings: To explain and to predict. Vol. 18, Journal of the Royal Society Interface. Royal Society Publishing; 2021.

17. Wei T, Simko V, Levy M, Xie Y, Jin Y, Zemla J. R package “corrplot”: Visualization of a Correlation Matrix. Statistician. 2017;56.

18. Stanton MC, Diggle PJ. Geostatistical analysis of binomial data: Generalised linear or transformed Gaussian modelling? Environmetrics. 2013;24(3).

19. Fuglstad GA, Simpson D, Lindgren F, Rue H. Constructing Priors that Penalize the Complexity of Gaussian Random Fields. J Am Stat Assoc. 2019;114(525).

20. Rue H, Martino S, Chopin N. Approximate Bayesian inference for latent Gaussian models by using integrated nested Laplace approximations. Journal of the Royal Statistical Society Series B: Statistical Methodology. 2009;71(2).

21. EPMM Working Group. Strategies toward ending preventable maternal mortality (EPMM) [Internet]. Geneva; 2015 [cited 2022 Feb 21]. Available from: https://www.who.int/reproductivehealth/topics/maternal_perinatal/epmm/en/
